# Lamotrigine Restores Impairments in Theta Rhythms and LTP as Early Biomarkers of Aβ_1-42_-Induced Hippocampal Network Dysfunction

**DOI:** 10.14336/AD.2025.0796

**Published:** 2026-10-04

**Authors:** Maciej Studzian, Bartosz Bobula, M. Bruce MacIver, Renata Bocian, Anna Kobrzycka, Bartosz Caban, Tomasz Kowalczyk, Jacek Grebowski, Sylwia Michlewska, Maciej M. Jankowski, Krzysztof Tokarski, Paulina Kazmierska-Grebowska

**Affiliations:** ^1^University of Lodz, Faculty of Biology and Environmental Protection, Department of Oncobiology and Epigenetics, 90-236 Lodz, Poland.; ^2^Institute of Medical Biology, Polish Academy of Sciences, Laboratory of Transcriptional Regulation, 93-232 Lodz, Poland.; ^3^Department of Physiology, Maj Institute of Pharmacology, Polish Academy of Sciences, 31-343 Krakow, Poland.; ^4^Neuroscience Institute, School of Medicine, Stanford University, Palo Alto, CA 94305, USA.; ^5^University of Lodz, Faculty of Biology and Environmental Protection, Department of Neurobiology, 90-236 Lodz, Poland.; ^6^Military Institute of Medicine - National Research Institute, 04-141 Warsaw, Poland.; ^7^University of Lodz, Faculty of Biology and Environmental Protection, Laboratory of Microscopic Imaging and Specialized Biological Techniques, 90-237 Lodz, Poland.; ^8^Gdansk University of Technology, Faculty of Electronics, Telecommunications and Informatics, Multimedia Systems Department, BioTechMed Center, 80-233 Gdansk, Poland

**Keywords:** Alzheimer’s disease;, hippocampus, amyloid β_1-42_;, theta rhythm, lamotrigine, HCN channels

## Abstract

Neuronal excitation/inhibition (E/I) imbalance, epileptiform activity, and synaptic dysfunction are present in individuals diagnosed with dementia, including sporadic Alzheimer’s disease (sAD), even at early stages. These abnormalities are associated with altered neuronal oscillatory activity, as seen in EEG recordings of sAD patients and transgenic rodents. Hippocampal theta oscillations are crucial for sensorimotor integration, memory consolidation, and network coordination, and depend on synaptic and ionic mechanisms, including the I*h* current, mediated by hyperpolarization-activated cyclic nucleotide-gated (HCN) channels. Lamotrigine (LTG), an HCN channel modulator, influences neuronal excitability and oscillations. Amyloid beta (Aβ) peptide, specifically Aβ_1-42_ form plays a key role in AD pathology, promoting hyperexcitability, synaptic dysfunction, and neurodegeneration, particularly in the hippocampus (HPC) and associative cortices. However, only a few electrophysiological studies have examined HPC theta in relation to early cognitive deficits in sAD animal models. Our first goal was to study the temporal progression of theta oscillations impairment following repeated unilateral intracerebroventricular (ICV) Aβ_1-42_ infusions in rats at 7, 14, and 21 days, *in vivo* and in *vitro*. Second, we assessed whether local LTG administration could restore theta oscillations and long-term potentiation (LTP) in this model. We found that: i) Aβ_1-42_ ICV infusions led to significant reductions in theta amplitude and power on days 7 and 14, with a complete loss by day 21; ii) LTG restored LTP and theta rhythms in terms of power and amplitude in HPC obtained from Aβ_1-42_-treated animals 14 days post-ICV infusion; iii) histological analysis confirmed neurodegeneration, Tau hyperphosphorylation, astrogliosis, increased number of parvalbimun positive (PV+) interneurons, and GluN2B receptor upregulation in HPC of Aβ_1-42_-treated animals 14 days post-ICV infusion. These findings suggest that hippocampal theta disruption may serve as an early biomarker of network dysfunction in sAD, and that LTG-mediated partial restoration of theta and LTP offers a potential early therapeutic strategy.

## INTRODUCTION

The β-amyloid peptide (Aβ) pathophysiology, remains a hallmark of Alzheimer’s Disease (AD) [1-3], and primarily impacts the hippocampus (HPC) and associative cortices [4, 5], resulting in neuronal hyperactivity [1, 6], synaptic and neuronal networks alterations [7-9], astrocytic dysfunction [10-12] and increased neuro-degeneration [13, 14]. These effects lead to alterations in hippocampal long-term synaptic depression (LTD) and potentiation (LTP) [15-17], that are neuronal substrates for memory formation [18], and also result in synaptic loss and dysfunction [19]. Due to their receptor-binding activity, particularly NMDA receptors (NMDARs), tau protein pathology induction, inflammation, and astrocytosis-triggering properties, as well as synaptotoxicity, Aβ soluble oligomers are considered the most neurotoxic form of Aβ peptides, observed in both humans and animal models of AD [3, 20-23].

While transgenic animal models, such as amyloid protein precursor (APP) and presenilin-1 (PS1) overexpressing models, are useful for studying genetic aspects of AD [24], sporadic AD (sAD), which accounts for about 90% of cases [25], requires distinct experimental approaches. Evidence suggests that Aβ-injected rodents closely replicate the main neuropathological symptoms observed in sAD patients [26-28]. Among diverse Aβ species Aβ_1-42_ variant is believed to be more toxic than other forms of Aβ peptide [20, 29, 30]. Faucher et al. [31] evidenced that repeated bilateral Aβ_1-42_ oligomers infusions into the dorsal HPC caused long-lasting working memory impairments, while Aβ_1-42_ oligomers injections in *stratum pyramidale* of CA1 HPC region caused a significant disruption in tonic inhibition, which may account for cognitive deficits during AD progression [32].

Early network dysfunction characterized by neuronal hyperactivity and altered oscillations resulting in the loss of neuronal synchrony is critical in AD development [30, 33-35] and often accompanied by seizures [7, 36]. This dysfunction may result from damage to inhibitory circuits [32, 37] including parvalbumin-positive (PV+) interneurons responsible for fine-tuning of neuronal network oscillations [38, 39] and astrocytes regulating glutamate and GABA extracellular levels [12]. In a genetic mouse model (APP/PS1 mice) of early AD stage Aβ oligomers were found to induce interneuronal hyperexcitability, and neuronal network dysfunction accompanied by memory deficits [40]. Consequently, network alterations, rather than protein deposition *per se*, could be key in the early AD pathogenesis [38, 41]. Neurons operate within interconnected networks, and cognitive decline induced by Aβ oligomers likely arises from disruptions in the fundamental mechanisms regulating neuronal network integrity [42]. Indeed, alterations in oscillatory network activity have been detected in the EEG of AD patients [43] as well as transgenic AD animals [44, 45]. Theta rhythms observed in the mammalian limbic cortex including HPC are a prime example of a field rhythmic oscillations, playing a key role in a variety of cognitive processes, including memory and attention [46, 47], decision-making, reception and integration of sensory stimuli [48]. Moreover, theta rhythms represent an essential physiological property frequently employed as a sensitive measure for examining neural network synchrony [47]. The link between theta oscillations and information processing is reinforced by findings that drugs enhancing memory increase theta oscillations, while those impairing memory formation decrease them [49].

A limited number of human studies suggest that theta rhythms could be considered as an early biomarker of cognitive decline in AD patients [50-53]. Changes in theta activity have been shown to precede amyloid plaque deposition, making theta a particularly attractive candidate for early-stage detection [5, 54-56]. In animal studies Peña et al. [49] evidenced for the first time that acute Aβ_25-35_ administration disrupts HPC field activity both *in vivo* and *in vitro* and reduces sensory-evoked theta-like activity in urethane-anesthetized rats. Aβ_1-42_ peptide administered intrahippocampally two weeks prior to EEG recordings significantly slowed hippocampal and cortical baseline EEG [57]. Interestingly Aβ_1-42_ peptide was found more potent in reduction of carbachol-induced theta *in vitro* than Aβ_25-35_ [58]. Those findings show a correlation between theta-related disruptions and Aβ peptide’s deleterious effect but did not study early stages of the pathology when therapeutic intervention could be most effective [59].

Many mechanisms by which Aβ peptides disrupt neural network excitability and oscillations remain unresolved, but stabilizing the excitatory/inhibitory (E/I) balance shows great promise for restoring hippocampal function in early AD-related dementia. In the realm of pharmacological interventions, lamotrigine (LTG) emerges as an up-and-coming tool [5, 60-62]. Lamotrigine is a new generation antiepileptic drug with a well-documented safety profile [62, 63] and anti-inflammatory properties [64]. A few studies have demonstrated that LTG prevented the recurrence of mood episodes in patients with bipolar disorder and reduced psychosis and memory loss in older individuals with dementia, including AD [65-67]. LTG mainly acts as an inhibitor of voltage-gated sodium channels, thereby attenuating the activity-dependent glutamate release from presynaptic terminals of excitatory neurons [5, 65, 68]. Additionally, apart from its ability to suppress a Ca^2+^-sensing cation current [69, 70] and to inhibit A-type potassium currents in cultured HPC neurons [71], LTG upregulates the I*h* current (hyperpolarization-activated cation current) mediated by hyperpolarization-activated cyclic nucleotide-gated (HCN) channels in the dendrites of hippocampal pyramidal neurons [72]. We have previously evidenced that LTG-dependent decrease in GABA_A_ receptor (GABA_A_Rs)-mediated fast transmission in CA3 neurons and depression of CA3 and CA1 pyramidal neuron excitability *in vitro* as well as increase in theta parameters *in vivo* was modulated by HCN channels [73]. Further research is needed to uncover how HCN channels influence E/I balance, network oscillations and synaptic plasticity, aiming to develop new therapies for preventing or treating cognitive impairments and dementias, particularly in the early stages of AD.

To date, no studies have directly investigated the effects of the most toxic Aβ_1-42_ oligomers on HPC theta rhythms shortly after intracerebral administration, nor explored disrupted theta as a potential early biomarker of neuronal functions impairment in sAD animal models. To address this, we examined the temporal relationship between theta rhythms quality and the elapsed time since repeated unilateral intracerebroventricular (ICV) infusions of Aβ_1-42_ oligomers (once daily for four consecutive days), conducting these investigations 7, 14-, and 21-days post-infusion and complementing them with histological verification. Intracerebroventricular administration of soluble Aβ oligomers offers a reliable rat model of early Alzheimer's pathology by avoiding mechanical damage, while preserving tissue integrity for electrophysiological recordings and reproducing key histopathological hallmarks of AD [74]. Accordingly, we assessed tau hyperphosphorylation along with alterations in astrocyte and PV+ interneuron representations across HPC subregions. We have also measured the expression of GluN2B receptors (GluN2BRs) as a key player implicated in Aβ-induced neuronal overactivity [75-78]. Additionally, given the lack of clinical trials on the use of LTG for early sAD treatment [62], we evaluate the effects of local LTG administration on hippocampal theta rhythms and LTP induction in Aβ_1-42_ ICV-infused rats with the ultimate goal of unlocking new treatment possibilities for sAD and/or other dementias.

## MATERIALS & METHODS

### Animals

All experimental procedures were performed in accordance with the European Communities Council Directive (86/609/EEC+2010/63/UE) and Local Ethical Commission in Poland (permission number: 1/LB122/2019). Experimental design and execution were performed according to the ARRIVE guidelines 2.0. Ninety-six male Wistar rats (Charles River Laboratories, Wilmington, MA, USA), 3 months old and weighing 250-300 g, were used in the experiments. Animals were housed in groups in standard cages with free access to standard laboratory diet and tap water. All animals were kept in a ventilated room, at a temperature of 22±1°C, under a 12-h light/12-h dark cycle (light between 8:00 a.m. and 8:00 p.m.). All efforts were made to minimize animal suffering, and the number of animals used.

### Surgery

The surgery was performed under general anaesthesia induced by intraperitoneal (IP) injection of a cocktail of ketamine [50 mg/kg] and xylazine [20 mg/kg]. During anaesthesia, the skull was placed in the stereotaxic apparatus for cannula implantation. The stainless-steel guide cannula with a small cap dummy cannula (22G, 6 mm length, Plastics One by Protech International, Inc., Boerne, TX, USA) was unilaterally positioned 1 mm above the right ventricle. Stereotaxic coordinates to Bregma were as follows: -0.6 mm anterior posterior; +1.7 mm lateral; -3.0 mm ventral [79]. The cannula was secured to the skull with four stainless steel screws and dental acrylic cement (Vertex, Vertex-Dental B.V., Netherlands). To avoid infections the postoperative wound was covered with crystalline penicillin (TZF, Poland). The recovery period lasted for 7 days before ICV infusions.

### Aβ_1-42_ preparation

Аβ_1-42_ peptide or its scrambled variant (SCR) were purchased from rPeptide (Athens, GA, USA). Peptides were first solubilized in hexafluoro-isopropanol (HFIP, Sigma Aldrich, USA), aliquoted into 1.5 ml low protein-binding Eppendorf tubes and the solvent was evaporated using a gentle stream of nitrogen. Immediately after peptide monomers were solubilized in buffer A (50 mM Tris-HCl pH=7.4, 1 mM EDTA), the protein concentration was determined with Pierce BCA protein assay (Thermo Fisher Scientific, Waltham, MA, USA) and adjusted to 0.3 mg/ml with buffer A. The final peptide solution was used for ICV infusions within 2 hours of preparation or stored in liquid nitrogen for later use (within one week of preparation; see [31] for more details). Transmission electron microscopy was used to visualize the aggregation of Аβ_1-42_ peptide and to assess the size of toxic oligomers in freshly prepared samples and at defined time points after thawing samples previously stored at -80°C or in liquid nitrogen (Supplementary Figs. 1-2). For this purpose, 10 µl of the samples were placed on 200 mesh grids with a carbon/formvar surface (TedPella Redding, CA, USA) and stained for 20 min using 2% uranyl acetate. Next, the grids were washed with deionized water. After drying at room temperature, the samples were examined using a JEM-1010 (JEOL, Tokyo, Japan) transmission electron microscope at 80 kV. Images were taken at a magnification of 40,000 × or 80,000 × and analyzed with conventional software. Alternatively, thioflavin T fluorescence assay was used to measure the aggregation process of Аβ_1-42_ and SCR peptides. The purchased peptides were first solubilized in HFIP to induce Аβ_1-42_ depolymerization. After the removal of the organic solvent, the peptides were resolubilized in PBS. To study oligomerization and fibril formation, thioflavin T (12 µM) was added to the samples immediately after peptide resolubilization to reach a final concentration of 40 µM. Fluorescence (Ex=450 nm, Em=482 nm) was measured using an EnVision microplate reader (PerkinElmer, Waltham, MA, USA) every 15 minutes for the first 21 hours (Supplementary Fig. 3).

### Aβ_1-42_ intracerebroventricular infusions

On infusion day the rat was gently restrained, and the small cap dummy cannula was replaced with a sterile infusion cannula (20 gauge, Plastics One by Protech International, Inc., Boerne, TX, USA) which extended 1 mm below the tip of the guide cannula. The peptide solution (Аβ_1-42_ oligomers or SCR) was administered with a 5 μl Hamilton microsyringe using an infusion pump (KD Scientific Inc., MA, USA). 4 μl of the respective solution was infused into the right cerebral ventricle at a rate of 0.5 μl/min. Following the infusion, the needle remained in place for the next 5 minutes to prevent solution reflux. The infusion cannula was retracted, and the cap dummy cannula was immediately replaced into the guide cannula. The infusions were repeated once per day for four consecutive days.

### Histopathological study

To confirm accurate delivery of Aβ_1-42_ oligomers into the lateral ventricles and to monitor the amyloid presence in HPC, brain tissue samples were collected on days 1, 7, and 14 following the final ICV infusion (one control animal infused with SCR and one animal infused with Aβ_1-42_ at each time point). For all other histological analyses, tissue samples were collected 14 days after the last ICV infusion. Following deep anesthesia with ketamine and xylazine, the rats (6 infused with SCR and 6 infused with Аβ_1-42_) were transcardially perfused, first with ice-cold saline (0.9% NaCl), and subsequently with 4% ice cold formaldehyde solution buffered in PBS (pH 7.4). Next, rats were decapitated, and whole brains were extracted and post-fixed for 48 h at 4°C in 4% PBS-buffered formaldehyde. After that, coronal sections (40 μm) were cut on a Leica VT1200 vibratome (Leica, Wetzlar, Germany) and stored in 4°C in PBS supplemented with 0.05% NaN_3_ until staining.

For H&E staining, selected brain sections including the infusion site (-0.80 mm from Bregma) as well as sections containing an undamaged cross-section of the third lateral ventricle (-0.30 mm from Bregma) were stained with Harris hematoxylin and eosin. Stained sections were mounted using Leica CV Mount and examined with a light microscope. Transmitted-light images were acquired with a semi-automated MICA microscope (Leica, Wetzlar, Germany), and the cross-sectional area of the third lateral ventricle was measured using LASX software (Leica, Wetzlar, Germany).

Immunohistochemical staining was performed using the free-floating technique. Selected brain sections were first blocked and permeabilized with buffer B (10% normal goat serum, 0.4% Triton X-100, PBS pH 7.4) for 1 hour. The sections were then incubated overnight at 4°C with primary antibodies (at least 300 µl of medium per section) diluted 1:100 in buffer (for details, see Supplementary Table 1). Following three washes in PBS with 0.05% Tween (PBST), the sections were incubated for 1 hour with respective secondary antibodies diluted 1:500 in Buffer B (Supplementary Table 1). Finally, the sections were washed three times with PBST, stained with 5 µM Hoechst 33342 for 15 minutes to visualize cell nuclei, and air-dried after being placed onto microscopy glass slides. Preparations were mounted using Leica CV Mount and examined by confocal microscopy.

### Confocal microscopy and histological data analysis

Images were obtained with a LSM 780 microscope (Carl Zeiss AG, Germany) equipped with either an EC Plan-Neofluar 10x/0.30 or a Plan-Apochromat 63x/1.40 objective. The InTune tunable excitation laser system or the 405 nm laser diode were used. Fluorescence of Hoechst 33342 stained cell nuclei was excited with the 405 nm laser and detected in the 415-480 range. Fluorescence of Alexa Fluor 488 was excited with a 490 nm laser line (InTune) and detected in the 500-560 nm range. Fluorescence of Alexa Fluor 594 nm was excited with a 595 nm laser (InTune) and detected in the 600-700 nm range. Tile scan mode was used to collect images of the entire HPC region. Images were stitched together using ZEN 2012 software (Carl Zeiss AG, Germany). Quantitative image analysis was performed with ZEN software (ZEN 2.1 Blue, Carl Zeiss AG, Germany). For the analysis of the GFAP signal, regions of interest for the respective HPC subareas (*subiculum*, CA1, CA2-CA3, DG) were placed in each image, and the percentage of GFAP-positive pixels (exceeding an intensity threshold) was calculated relative to the total number of pixels in the given area. For the analysis of PV+ cell bodies, regions of interest were defined as described above, and in three sections per brain containing the HPC (separated by approximately 400 µm), the number of objects identified as PV+ interneuron cell bodies was counted. To analyze GluN2BRs expression, the average fluorescence intensity was calculated for each section within a 5×5 field of view (630× magnification) located in the *stratum radiatum* of CA1. The data were tested for normality using the Shapiro-Wilk test and for homogeneity of variance using Levene’s test. Intergroup statistical differences were evaluated using an unpaired t-test. Statistical significance was defined as p ≤ 0.05.

## *In vivo* and *in vitro* electrophysiology

### Hippocampal local field potentials (LFPs) recording in vivo

Hippocampal LFPs recordings were conducted on days 7, 14, or 21 after the last ICV infusion of SCR or Аβ_1-42_ oligomers in anesthetized rats. Additionally, in animals previously subjected to ICV infusion, LTG (4 µg/µl) was administered intrahippocampally 14 days later, to assess the effect of a single-dose treatment on hippocampal theta rhythms. To implant the recording electrodes, the animals were positioned in a stereotactic frame ensuring that the plane between Bregma and Lambda was horizontally aligned. An uninsulated tungsten wire was inserted into the cortex, 2 mm anterior to Bregma, and served as an indifferent electrode. A unipolar tungsten microelectrode, with impedance ranging from 0.3 to 0.9 MΩ, was placed into the right dorsal HPC within the *stratum lacunosum-moleculare*, located at coordinates 3.2 mm posterior to Bregma, 2.2 mm lateral to the midline, and 2.6-2.8 mm ventral to the dural surface [79]. The hippocampal LFPs were recorded using an AC amplifier (P-511, Grass-Astromed, USA) with a high-pass filter set at 1 Hz, and a low-pass filter set at 0.3 kHz. The LFPs were digitized *via* a Micro1401 interface (Cambridge Electronic Design, Milton, UK) at a sampling frequency ranging from 0 Hz to 50 Hz, divided into 64 bins, and then displayed and stored on a computer hard disk for subsequent offline analysis.

### Hippocampal slice preparation and local field potentials recording in vitro

Hippocampal LFPs recordings *in vitro* were conducted on days 7, 14, or 21 after the last ICV infusion of SCR or Аβ_1-42_ oligomers. Each animal was initially anesthetized with isoflurane (Aerrane, Baxter, UK) and then decapitated. The brain was quickly removed and placed in cold (3-5°C) and oxygenated (95% O_2_+5% CO_2_) artificial cerebrospinal fluid (ACSF) (121 mM NaCl, 5 mM KCl, 2.5 mM CaCl_2_, 1.25 mM KH_2_PO_4_, 1.3 mM MgSO_4_, 26 mM NaHCO_3_, and 10 mM glucose, pH 7.4). ACSF was freshly prepared before each experiment using prefiltered and deionized water. Hippocampal slices (500 μm) were prepared from both hippocampi using a Stoelting tissue slicer (Wood Dale, IL, USA) and preincubated in oxygenated ACSF at approximately 20°C for 45 min post-dissection. Subsequently, the slices were transferred to a gas-liquid interface recording chamber and placed on nylon mesh, where they were constantly perfused with oxygenated and prewarmed (35°C) ACSF at a slow flow rate of 1 ml/min for 45 min. Hippocampal LFPs *in vitro* were elicited by perfusing with carbachol (carbamoyl-choline chloride-CCH; Sigma-Aldrich, St. Louis, MO, USA), which was dissolved in ACSF to a concentration of 50 µM. Extracellular LFP measurements were conducted using glass recording electrodes (3-5 MΩ, Kwik-Fil capillaries, WP Instruments, Sarasota, FL, USA) relative to the ground. The electrodes were positioned in the CA3c subfield of HPC using a motorized micropositioner (IVM-1000; Scientifica, Uckfield, UK). The recorded signals were filtered (1 to 300 Hz band-pass), amplified (1000×) with a P-511 preamplifier (Grass-Astromed, West Warwick, RI, USA), and saved onto a computer hard drive *via* a CED-1401 ADC converter with a sampling rate of 1000 Hz (Cambridge Electronic Design, Milton, UK).

### In vivo and in vitro electrophysiology data analysis

The waveform data obtained from *in vivo* and *in vitro* experiments were analyzed offline using Spike 2.7 software (Cambridge Electronic Design, Milton, UK). The detailed analysis of *in vitro* recordings covered three 20-s samples (technical repetitions) starting at 40, 70, and 100 s of each recording, respectively, which were selected from each 2-min LFP fragment. For each *in vivo* experiment, ten 2-s samples of hippocampal LFPs were used to calculate the mean values of basic theta parameters. Selected epochs of hippocampal LFPs were subjected to power/frequency (fast Fourier transform-FFT) analysis. FFT size was 128 (0.03072 s), Hanning window, frequency from 0-50 Hz in 64 bins, resolution 0.7813 Hz. The mean peak-to-peak amplitude of theta waves was measured directly from the theta waveforms. Hippocampal theta epochs were defined by peaks in the power spectra within the 3-6 Hz frequency band *in vivo* and 6-12 Hz *in vitro* and confirmed by visual inspection of the raw LFP traces. Spectrograms (top dB 96 × pixel 1, Hanning window, range dB 96, block size 1024) were produced from hippocampal LFPs recorded over 15 s. The statistical calculations were performed using STATISTICA software (v.13.1., Cloud Software Group, USA). The results are shown as the median and quartile range (lower-upper quartile, 25-75%). The data were tested for normal distribution with the Shapiro-Wilk test and for variance homogeneity with Levene’s test. Statistical significance between groups was evaluated using the appropriate ANOVA (two-way ANOVA or repeated-measures ANOVA), followed by the Bonferroni *post hoc* test. Statistical significance was defined as p ≤ 0.05.

### Long-term potentiation recording and data analysis

Animals previously subjected to Аβ_1-42_ or SCR ICV infusion were anesthetized with isoflurane (Aerrane, Baxter, UK) on the 14’th day after the infusion and HPC slices (380 μm thick) were cut using a vibrating microtome (Leica VT1000s, Germany) in N-methyl-D-glucamine (NMDG)-based ACSF containing: 92 mM NMDG, 2.5 mM KCl, 0.5 mM CaCl_2_, 10 mM MgSO_4_, 1.2 mM NaH_2_PO_4_, 30 mM NaHCO_3_, 20 mM HEPES, 25 mM glucose, 5 mM sodium ascorbate, 2 mM thiourea, 3 mM sodium pyruvate, pH=7.3. Slices were then incubated at 32°C for 25 min while gradually introducing NaCl (the ‘Na spike-in’ method adopted from Ting et al. [80]). When the ‘Na spike-in’ protocol was completed, slices were transferred to ACSF containing: 132 mM NaCl, 26 mM NaHCO_3_, 2 mM KCl, 2.5 mM CaCl_2_, 1.3 mM MgSO_4_, 1.25 mM KH_2_PO_4_, and 10 mM D-glucose, bubbled with 95% O_2_+5% CO_2_ at 2.5 ml/min (32±0.5°C), in a filled incubation chamber with or without LTG at a concentration of 50 μM for 60 min. After the incubation, a single slice was transferred to the interface recording chamber. An ACSF-filled glass microelectrode (~1 MΩ) was placed in the *stratum radiatum* to record the field excitatory postsynaptic potentials (fEPSPs). A concentric Pt-Ir stimulating electrode (FHC Inc., Bowdoin, ME, USA) was placed in the *stratum radiatum*. Stimuli (duration: 0.2 ms) were applied at 0.016 Hz using a constant-current stimulus isolation unit (WP Instruments, USA). Responses were amplified (Axoprobe 1A, Axon Instruments, USA), A/D converted at 10 kHz, and stored using a Micro1401 interface and Signal 4 software (Cambridge Electronic Design, Milton, UK). A stimulus-response curve was obtained for each slice. To obtain the curve, stimulation intensity was gradually increased stepwise (21 steps; 0-100 μA) and one response was recorded at each stimulation intensity. Next, stimulation intensity was adjusted to evoke a response of 30% of the maximal fEPSP slope, and three responses to paired pulses (50 ms interpulse interval) were recorded and averaged. For the induction of LTP, a theta burst stimulation protocol (TBS) was used. TBS consisted of five trains of stimuli at 5 Hz, each composed of 5 pulses at 100 Hz, repeated 3 times every 15 s. The stimulus-response curves were fit with the Boltzmann equation using Graphpad Prism software 10.4.0 (USA). The amount of LTP was determined as an average increase in the amplitude of field potentials recorded between 45 and 60 min after TBS, relative to baseline. The results are shown as the median and quartile range (lower-upper quartile, 25-75%). Statistical analysis was carried out using two-way ANOVA followed by Tukey’s *post hoc* test (GraphPad Prism 10.4.0. software, USA). Statistical significance was defined as p ≤ 0.05.

## RESULTS

### Aβ_1-42_ ICV infusions induce a progressive decline in hippocampal theta amplitude and power *in vivo*

We first sought to characterize the temporal dynamics of theta oscillations parameters on days 7, 14, and 21 following repeated unilateral ICV infusions of Aβ_1-42_ or SCR peptide. By day 21 in the Aβ_1-42_ group, theta oscillations were not observed in hippocampal LFP recordings (Fig. 1A-B). Therefore, only the data obtained on days 7 and 14 were statistically analyzed using two-way ANOVA. To examine the main effects of Aβ_1-42_ and SCR ICV infusions along with post-infusion time on theta parameters, we measured theta amplitude, power, and frequency across different time points. We observed that both theta amplitude and power were significantly reduced following Aβ_1-42_ ICV administration, irrespective of the post-infusion time (amplitude: F_(1,16)_=807.80, p≤0.0001; power: F_(1,16)_=2417.72, p≤0.0001; Fig. 1A). Further analysis showed a significant decrease in theta amplitude and power on day 14 compared to day 7 post-infusion (amplitude: F_(1,16)_=4.91, p=0.0419; power: F_(1,16)_=29.61, p≤0.0001; Fig. 1A). This effect was modified by an interaction between infusion type and post-infusion time, as theta amplitude and power levels differed between groups across time points (amplitude: F_(1,16)_=2.34, p=0.0002; power: F_(1,16)_=34.80, p≤0.0001; Fig. 1A). Bonferroni *post hoc* test confirmed that both theta amplitude and power were significantly reduced in animals subjected to Aβ_1-42_ ICV infusions at each time point compared to SCR controls (amplitude: SCR^7^ *vs.* Aβ_1-42_^7^, p≤0.0001; SCR^14^ *vs.* Aβ_1-42_^14^, p≤0.0001; power: SCR^7^ *vs.* Aβ_1-42_^7^, p≤0.0001; SCR^14^ *vs.* Aβ_1-42_^14^, p≤0.0001; Fig. 1A). Notably, *post hoc* analysis also indicated a significant reduction in theta amplitude and power on day 14 compared to day 7 in Aβ_1-42_-infused animals (amplitude: Aβ_1-42_^7^ *vs.* Aβ_1-42_^14^, p=0.0009; power: Aβ_1-42_^7^ *vs.* Aβ_1-42_^14^, p≤0.0001; Fig. 1A), a change absent in SCR-infused controls (amplitude: SCR^7^ *vs.* SCR^14^ p=0.5694; power: SCR^7^ *vs.* SCR^14^ p=1.0000). No effects of Aβ_1-42_/SCR ICV infusions (F_(1,16)_=0.01, p=0.9282) and post-infusion time (F_(1,16)_=3.19, p=0.0932), nor their interaction (F_(1,16)_=0.17, p=0.6858) on theta frequency were observed (Fig. 1A). These findings demonstrate that repeated Aβ_1-42_ ICV infusions significantly reduce theta amplitude and power in hippocampal LFP recordings, with these effects becoming more pronounced over time (Fig. 1A-B).

**Figure 1.**
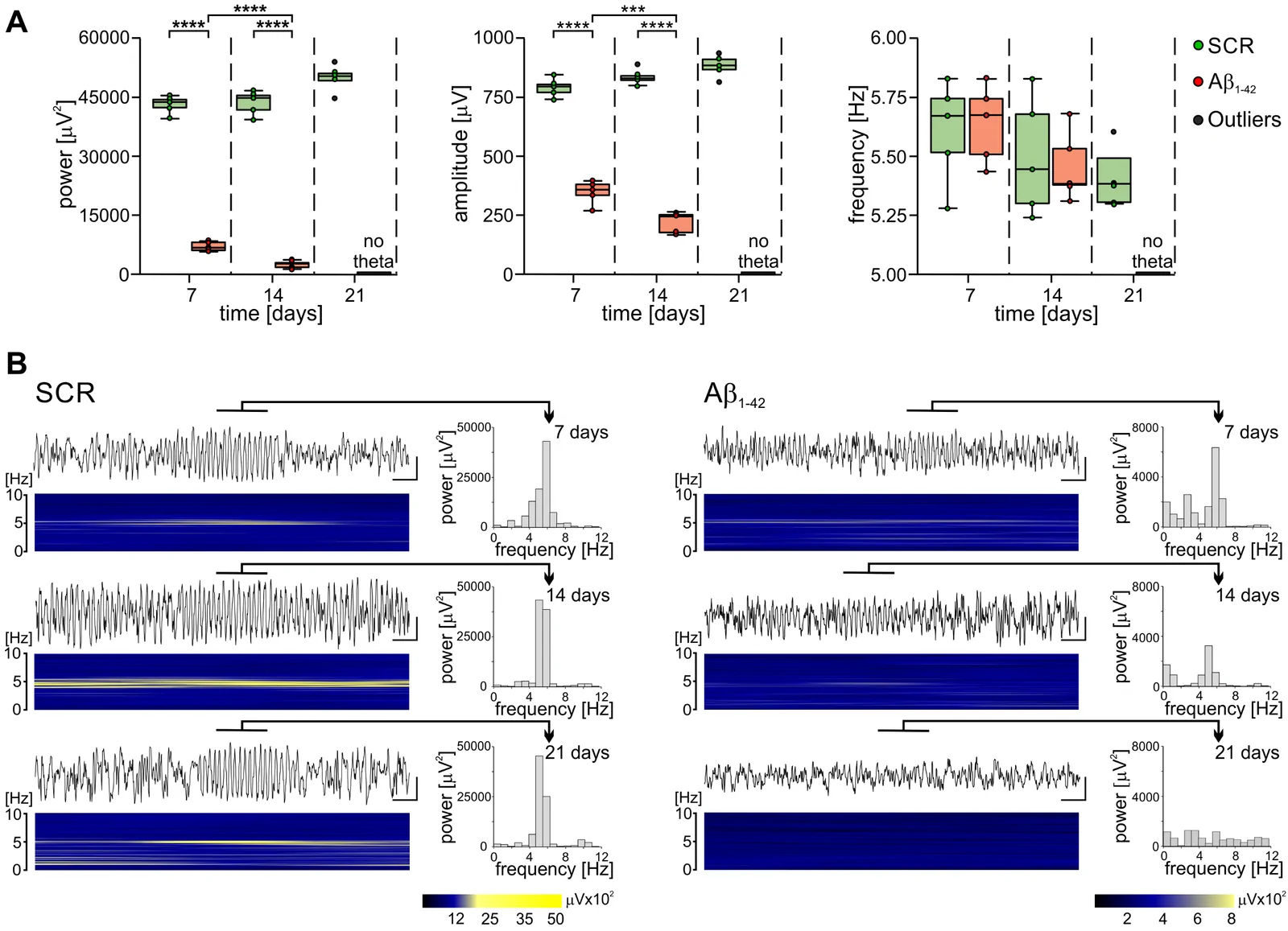
**Attenuating effect of intracerebroventricular (ICV) infusions of Aβ_1-42_ oligomers (Aβ_1-42_) *vs***. scrambled Aβ_1-42_ peptide (SCR) on hippocampal (HPC) theta oscillations recorded in anesthetized rats (n=5 per group). (**A**) Alterations in power and amplitude, but not frequency of HPC theta rhythms on day 7, 14, and 21 following ICV infusion were observed. Data are presented as the median (horizontal line) with lower and upper quartile ranges (25-75%, box) with whiskers for minimum and maximum. Significant differences were assessed using two-way ANOVA followed by the Bonferroni *post hoc* test (*p≤0.05, **p≤0.01, ***p≤0.001, ****p≤0.0001). (**B**) The power-frequency spectrograms taken from 15 s HPC field potentials recordings in animals pretreated with SCR peptide (left panel) and Aβ_1-42_ oligomers (right panel) in three time points: 7, 14, 21 days post-ICV infusion. Arrows indicate FFT histograms calculated from HPC local field potential samples (2s) recorded in CA1 field of HPC *in vivo*. Calibration: 1 s and 500 μV for SCR; 1 s and 250 μV for Aβ_1-42_.

### Aβ_1-42_ ICV infusions induce a progressive decline in hippocampal theta amplitude, power, and frequency *in vitro*

To complement our *in vivo* findings, we sought to investigate the temporal effects Aβ_1-42_ infusion on isolated HPC tissue to better understand the direct impact of Aβ_1-42_ on intrinsic oscillatory neural network properties, independent of external inputs. Consistent with prior findings, theta oscillations were absent in HPC slices obtained from Aβ_1-42_ ICV-infused animals 21 days post-infusion (Fig. 2A-B), thus directing statistical two-way ANOVA analysis to the data from days 7 and 14 post-infusion. Evaluating the main effects of Aβ_1-42_/SCR infusion and post-infusion time, we found that all CCH-induced theta parameters were significantly reduced in HPC slices obtained from Aβ_1-42_ ICV-infused animals, regardless of the post-infusion day (amplitude: F_(1,59)_=390.92, p≤0.0001; frequency: F_(1;61)_=1450.43, p≤0.0001; power _F(1,60)_=371.17, p≤0.0001; Fig. 2A). Additionally, theta frequency and power were significantly reduced in HPC slices on day 14 compared to day 7 post-infusion (frequency: F_(1,61)_=117.31, p≤0.0001; power: F_(1,60)_=120.33, p≤0.0001; Fig. 2A) and changes in amplitude trended similarly but were not statistically significant (F_(1,59)_=3.41, p=0.0697; Fig. 2A). Consistent with *in vivo* data, the interaction of infusion type and post-infusion time influenced these outcomes, with all CCH-induced theta parameters differing across groups depending on the time after infusion (amplitude: F_(1,59)_=14.58, p=0.0003; frequency: F_(1,61)_=68.58, p≤0.0001; power: F_(1,60)_=45.06, p≤0.0001; Fig. 2A). Bonferroni *post hoc* test confirmed a significant reduction in all measured CCH-induced theta parameters in HPC slices obtained from Aβ_1-42_ ICV-infused animals at both time points (amplitude: SCR^7^ *vs*. Aβ_1-42_^7^, p≤0.0001; SCR^14^ *vs*. Aβ_1-42_^14^, p≤0.0001; frequency: SCR^7^ *vs*. Aβ_1-42_^7^, p≤0.0001; SCR^14^ *vs*. Aβ_1-42_^14^, p≤0.0001; power: SCR^7^ *vs*. Aβ_1-42_^7^, p≤0.000; SCR^14^ *vs*. Aβ_1-42_^14^, p≤0.0001; Fig. 2A). Further *post hoc* analysis revealed a significant reduction in amplitude, frequency, and power of CCH-induced theta oscillations on day 14 relative to day 7 in HPC slices obtained from Aβ_1-42_-infused animals (amplitude: Aβ_1-42_^7^ *vs.* Aβ_1-42_^14^, p=0.0075; frequency: Aβ_1-42_^7^ *vs.* Aβ_1-42_^14^, p≤0.0001; power: Aβ_1-42_^7^ *vs.* Aβ_1-42_^14^, p≤0.0001; Fig. 2A).

**Figure 2.**
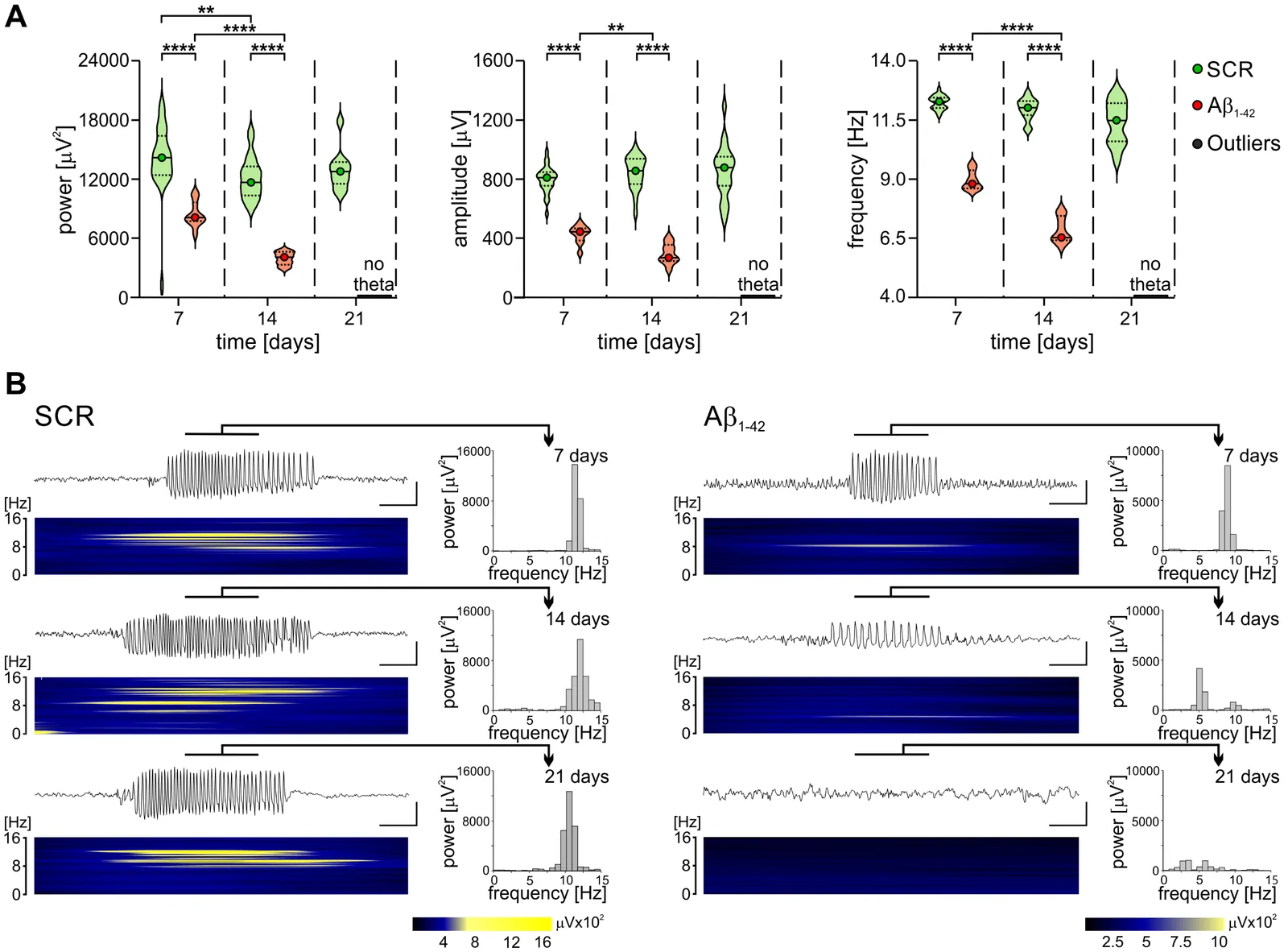
**Attenuating effect of intracerebroventricular (ICV) infusions of Aβ_1-42_ oligomers (Aβ_1-42_) *vs***. scrambled Aβ_1-42_ peptide (SCR) on *in vitro* recorded hippocampal (HPC) theta oscillations. (**A**) Alterations in power, amplitude, and frequency of HPC theta rhythms on day 7, 14, and 21 (n=25, n=21, and n=20 representing active HPC slices in SCR group; n=10, n=9, and n=0 representing active HPC slices in Aβ_1-42_ group, respectively) following ICV drug infusion were observed. Data are presented as violin plots with the median (horizontal line), with lower and upper quartile ranges (25-75%, dotted line). Significant differences were assessed using two-way ANOVA followed by the Bonferroni *post hoc* test (*p≤0.05, **p≤0.01, ***p≤0.001, ****p≤0.0001). (**B**) The power-frequency spectrograms taken from 10 s field potential recordings in HPC slices obtained from animals pretreated with scrambled SCR peptide (left panel) and Aβ_1-42_ oligomers (right panel) in three time points: 7, 14, 21 days post-infusion. Arrows indicate FFT histograms calculated from HPC field potential samples (2s) recorded in the CA3c field of HPC slices. Calibration: 1 s and 500 μV for SCR; 1 s and 250 μV for Aβ_1-42_.

Notably, a decrease in power was also observed in HPC slices obtained from SCR-infused animals (SCR^7^ vs. SCR^14^, p=0.0014), while amplitude and frequency remained unaffected (amplitude: SCR^7^ vs. SCR^14^, p=0.4663; frequency: SCR^7^ vs. SCR^14^, p=0.1310; Fig. 2A). These findings corroborate our *in vivo* observations, showing that Aβ_1-42_ ICV infusion progressively disrupts HPC theta oscillations. Significant reductions in theta amplitude, frequency, and power by day 14, and their absence by day 21 (Fig. 2A-B), highlight the time-dependent neurotoxic effects of Aβ_1-42_ oligomers.

**Figure 3.**
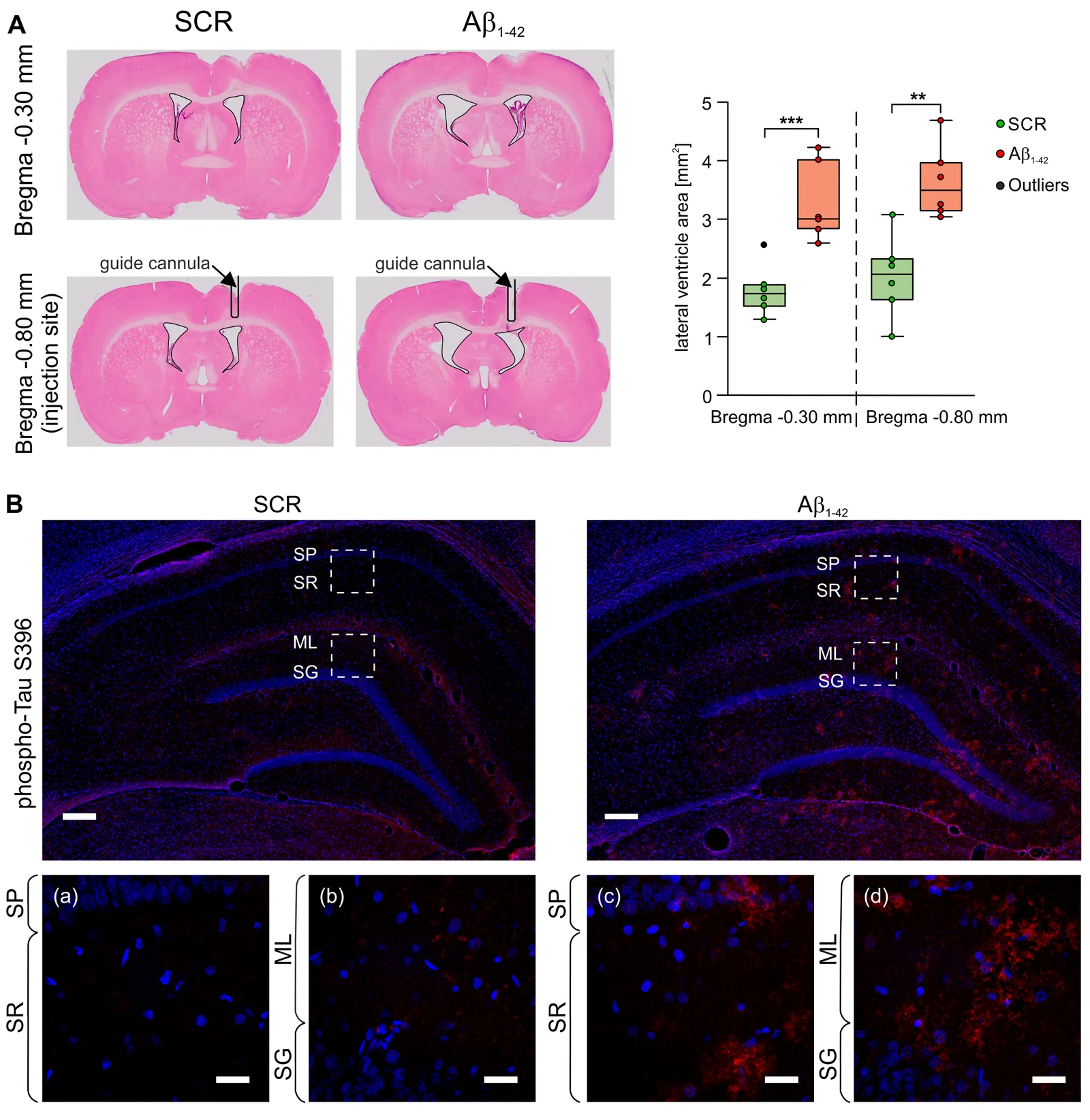
**Lateral ventricles enlargement and Tau protein hyperphosphorylation in the hippocampus (HPC) as an effect of Aβ_1-42_ intracerebroventricular (ICV) infusion (n=6 per group)**. (**A**) Neurodegeneration evidenced by enlargement of the lateral ventricles after Aβ_1-42_ infusion *vs.* SCR controls as shown in coronal H&E-stained sections, with ventricle areas and injection-site scarring outlined in black (left panel). Analysis of lateral ventricle size demonstrated a significant enlargement in Aβ_1-42_-treated animals compared to SCR controls (right panel). Intergroup statistical differences were evaluated using an unpaired t-test (*p≤0.05, **p≤0.01, ***p≤0.001, ****p≤0.0001). Data are presented as the median (horizontal line) with lower and upper quartile ranges (25-75%, box) with whiskers for minimum and maximum. (**B**) Elevated pTau (Ser396) immunoreactivity in HPC sections following Aβ_1-42_ infusion (red); counterstained cell nuclei (blue; approx. Bregma -3.80 mm). Upper images: overview of pTau distribution within the HPC subregions after SCR and Aβ_1-42_ ICV infusion (scale bar: 200 µm). Lower images: a high-magnification (scale bar: 20 µm) of the squared areas of HPC subregions; *stratum pyramidale* (SP) and *stratum radiatum* (SR) of CA1 and *molecular layer* (ML) and *stratum granulosum* (SG) of DG, indicating multiple pTau aggregates following Aβ_1-42_ICV infusion (c, d) compared to SCR ICV (a, b) controls.

### Soluble Aβ_1-42_ oligomers trigger aberrant tau phosphorylation, astrogliosis, increased PV+ interneurons density, and increased expression of GluN2B receptors in HPC 14 Days post-Aβ_1-42_ ICV infusion

Given the marked changes in HPC theta on day 14 post-Aβ_1-42_ ICV infusion, we examined key histopathological alterations at this time point compared to SCR-infused controls. Brains were coronally sectioned to assess ventricular changes following peptide infusion into the lateral ventricle. Two regions were selected for analysis: one at the injection site and another 0.5 mm anterior, where the ventricle is still clearly visible and cannula-induced scarring is minimal. We measured the area occupied by the lateral ventricles in each section as a proxy for total ventricle volume (Fig. 3A). In Aβ_1-42_ ICV-infused rats, the lateral ventricle area in both analyzed brain regions was nearly twice as large on average compared to controls infused with SCR (Bregma-0.80 mm, p=0.0018; Bregma-0.30 mm, p=0.0010; Fig. 3A). The total brain section area in each region was similar between the experimental groups indicating that the ventricular enlargement in Aβ_1-42_-infused rats reflects significant atrophy of surrounding tissues, consistent with the neurodegeneration characteristic of AD development. Next, we analyzed changes in brain sections containing the HPC. First, we attempted to visualize Aβ_1-42_ oligomers/fibrils using immunostaining. Interestingly, at this time point (14 days post-ICV infusion), no amyloid signal was detected (Supplementary Materials, Fig. S4). To confirm accurate delivery of Aβ_1-42_ oligomers into the lateral ventricles and to monitor the amyloid presence in HPC, we analyzed additional tissue samples collected on days 1 and 7 after the last infusion. At day 1 post-infusion, amyloid was detected both near the site of Aβ_1-42_ administration (around the ventricular ependymal cells) and in the HPC. By day 7, the presence of amyloid in the HPC could still be confirmed by immunostaining, although the amount of detectable Aβ_1-42_ was already reduced (Supplementary Materials, Fig. S4). In parallel, we assessed another well-established hallmark of AD, the level of Tau protein hyperphosphorylation (Fig. 3B). Immunostaining with an antibody against Tau phosphorylated at serine 396 revealed a marked increase in Tau phosphorylation in the HPC of Aβ_1-42_ ICV-infused rats on 14 day post-infusion. Notably, high levels of S396 hyperphosphorylated Tau (pTau) were localized to the DG and CA1 regions of the HPC in Aβ_1-42_ ICV-infused animals. High-magnification imaging further showed that, while pTau was present in some cells of SCR ICV-infused rats, Tau in Aβ_1-42_ ICV-infused rats was heavily hyperphosphorylated, forming multiple aggregates in the *stratum radiatum* of CA1 and the molecular layer of DG (Fig. 3B).

To further investigate the causes of the observed alterations in HPC physiology in Aβ_1-42_ ICV-infused animals, we performed immunostaining for neurons using an antibody against NeuN protein and for astrocytes using an antibody against GFAP protein (Fig. 4A-B). Consistent with the neurodegeneration observed around the ventricles in Aβ_1-42_-treated rats, the overall number of neuronal cell bodies (NeuN-positive regions) was reduced compared to SCR controls. This reduction was particularly evident in the granular cell layer of the DG and pyramids in CA2-CA3 regions, although some neurodegeneration was also observed in the CA1 and *subiculum* regions (Fig. 4B, white arrows). Additionally, we observed pronounced astrogliosis, characterized by both astrocytic hypertrophy and increased astrocyte density in all HPC regions in Aβ_1-42_ ICV-infused rats. The GFAP signal in the HPC was significantly higher in Aβ_1-42_ ICV-infused rats compared to SCR controls (Fig. 4A). In the CA1 and DG regions, the GFAP-positive area was increased by approximately 80% (CA1, p=0.0007; DG, p=0.0008), while in the CA2-CA3 and *subiculum* regions, it was more than doubled (CA2-CA3, p=0.0002; *subiculum*, p=0.0002; Fig. 4A). High-magnification imaging further revealed marked hypertrophy of astrocytes in several HPC subregions, particularly in the *stratum radiatum* and *stratum lacunosum moleculare* of CA1, as well as the molecular layer of DG in Aβ_1-42_ ICV-infused rats (Fig. 4B). These observations suggest that, at this stage, the effects of Aβ_1-42_ oligomer infusion on HPC function are likely not due to the deposition of amyloid plaques, but may instead be driven by other mechanisms, such as neuroinflammation and tau hyperphosphorylation. This aligns with the hypothesis that early-stage AD pathology is mediated by soluble forms of Aβ, which can exert neurotoxic effects even in the absence of detectable plaque deposition.

To investigate other potential cellular causes underlying the observed electrophysiological effects, we performed immunostaining for parvalbumin, a molecular marker of PV+ interneurons crucial for maintaining an adequate E/I balance in the HPC. Since HPC contains a limited number of PV+ interneurons, three different coronal sections containing the HPC were stained for each rat (Fig. 5A). In each section, we counted the number of PV+ interneurons in the respective subregions. Representative images from brain sections at -3.80 mm from Bregma revealed a visibly higher number of PV+ interneurons in Aβ_1-42_ ICV-infused rats compared to SCR controls (Fig. 5A). The most pronounced difference was observed in the CA1 region, since Aβ_1-42_-treated rats displayed more than twice the number of PV+ interneurons as SCR-treated rats (p≤0.0001; Fig. 5A).

**Figure 4.**
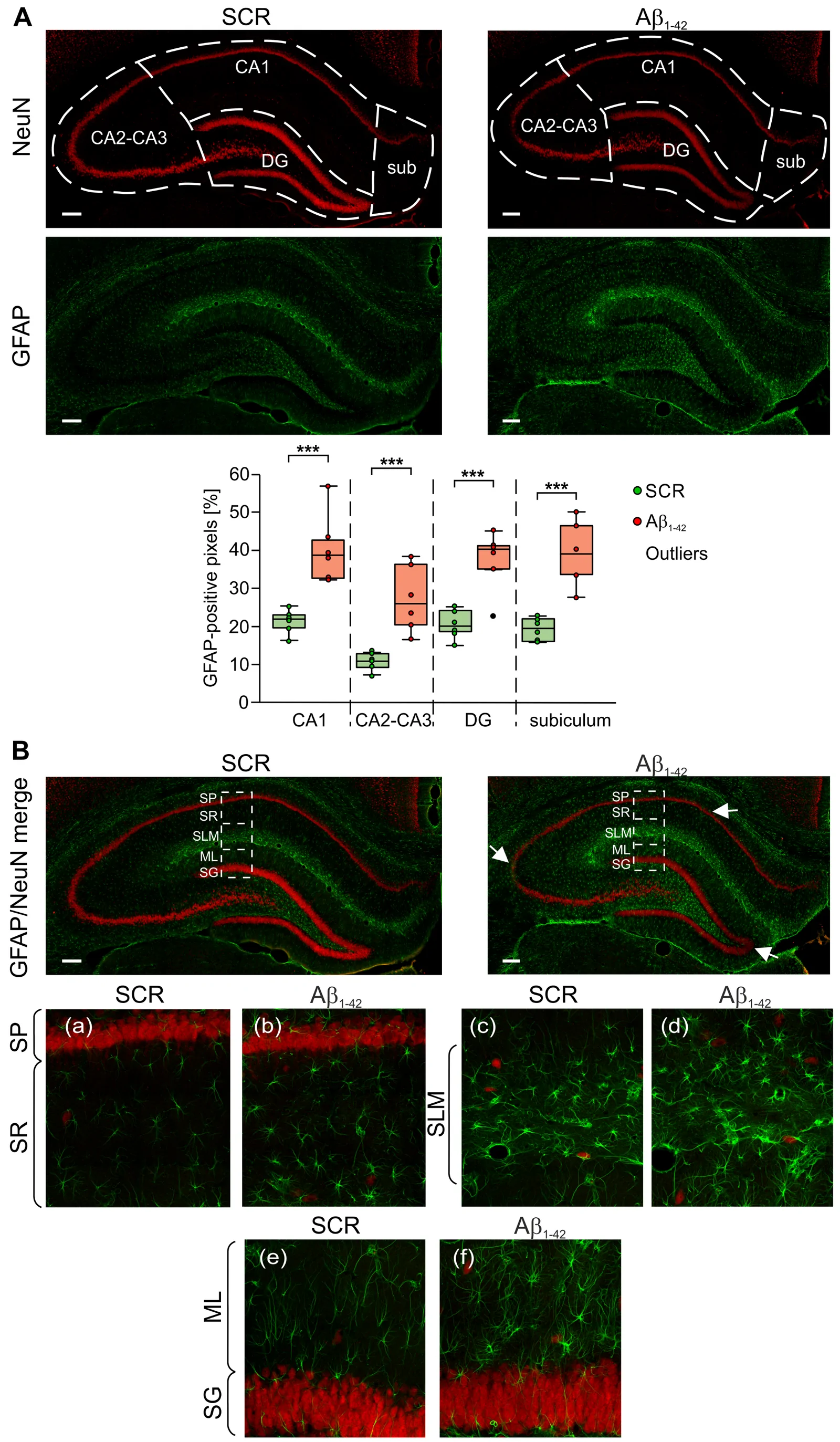
**Astrogliosis and neuro-degeneration in hippocampal (HPC) subregions as an effect of Aβ_1-42_ intracerebroventricular (ICV) infusion (n=6 per group)**. (**A**) Markers of neuronal cell bodies (NeuN, red) and astrocytes (GFAP, green) were immunostained in selected rat brain sections (approx. Bregma -3.80 mm) after SCR and Aβ_1-42_ ICV infusion. Quantification of the percentage area occupied by astrocytes revealed a significantly increased GFAP-positive signal in Aβ_1-42_-treated animals compared to SCR-treated controls in specific HPC subregions (marked with dashed white lines in upper panel A). Intergroup statistical differences were evaluated using an unpaired t-test (*p≤0.05, **p≤0.01, ***p≤0.001, ****p≤0.0001). Data are presented as the median (horizontal line) with lower and upper quartile ranges (25-75%, box) with whiskers for minimum and maximum. (**B**) Upper images: overview of NeuN and GFAP distribution within the CA1 and DG layers (scale bar: 200 µm). Areas with prominent neurodegeneration in the CA1 and CA2 pyramidal (*stratum pyramidale;* SP) and DG granular (*stratum granulosum;* SG) layers after Aβ_1-42_ *vs.* SCR ICV infusion are indicated by white arrows. Lower images: a high-magnification (scale bar: 20 µm) images of the squared areas of HPC subregions display astrocytosis and astrocyte hypertrophy in *stratum radiatum* (SR), *stratum lacunosum-moleculare* (SLM) of CA1, and *molecular layer* (ML) of DG subregions after Aβ_1-42_ (b, d, f respectively) *vs*. SCR ICV (a, c, e respectively) infusion.

Significant increases were also observed in the CA2-CA3 regions (p≤0.0001) and *subiculum* (p=0.0012; Fig. 5A). In contrast, the number of PV+ cells in the DG region remained the same both in SCR and Aβ_1-42_ ICV-infused animals (p=0.0963; Fig. 5A), albeit it should be noted that the DG naturally contains very few PV+ interneurons [81]. High-magnification images revealed that in the CA1 region of Aβ_1-42_ ICV-infused rats, there was not only an increased number of PV+ cell bodies localized in the cellular layer but also evidence of hypertrophy and much more densely packed network of perisomatic and dendritic connections compared to SCR-treated controls (Fig. 5A). Finally, we immunoassayed for the GluN2BRs and observed a significantly higher expression in the *stratum radiatum* of CA1 Aβ_1-42_ ICV-infused rats compared to SCR controls (p≤0.0001; Fig. 5B). Notably, increased fluorescence intensity of this receptor was detected in both the perisomatic regions and the dendritic shafts extending from the apices of pyramidal cells (Fig. 5B). These results suggest that Aβ_1-42_ ICV infusion induces significant upregulation of excitatory signaling and alterations in inhibitory circuitry, potentially reflecting an early-stage compensatory mechanism.

**Figure 5.**
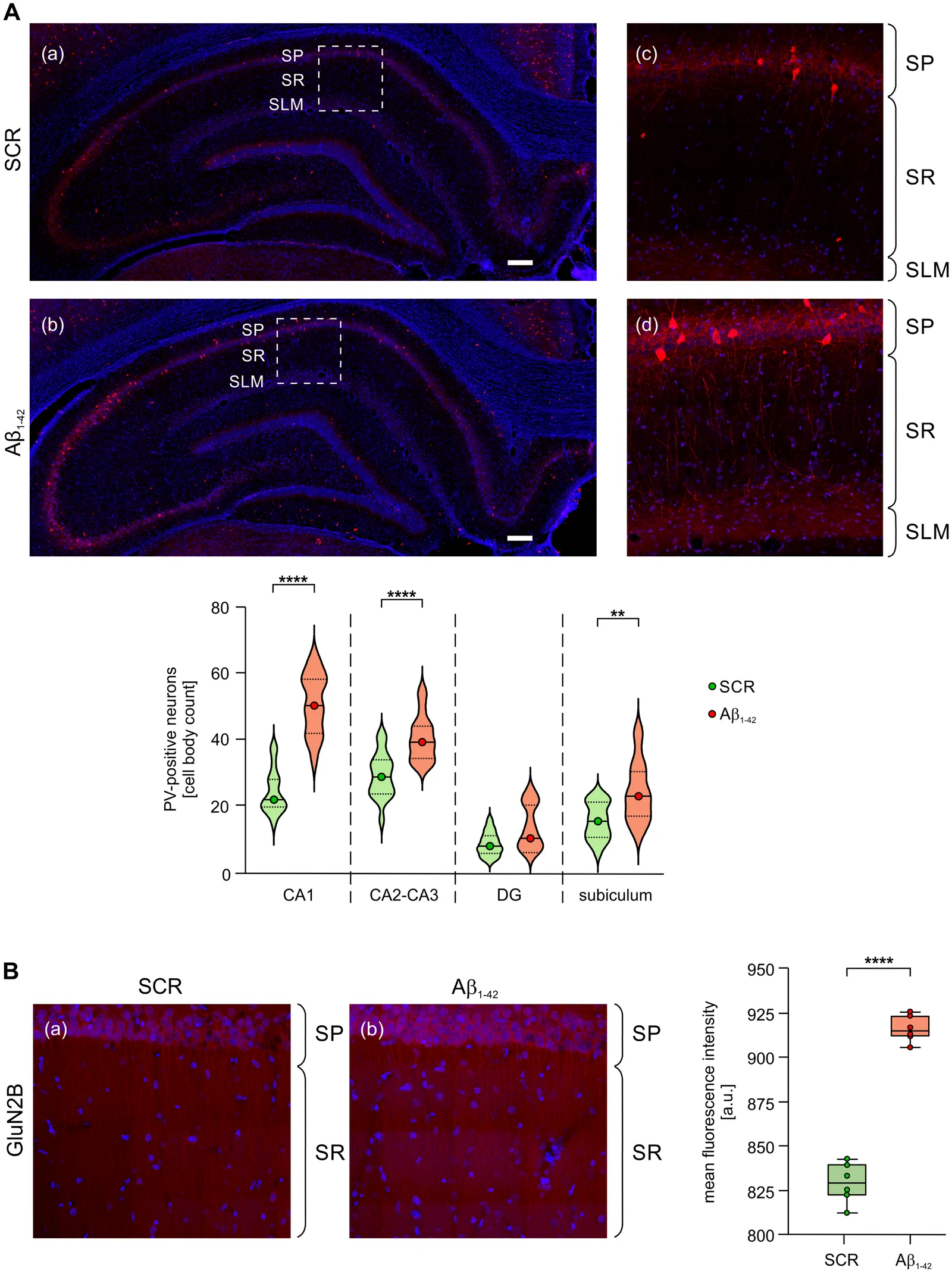
**Increased number of PV-positive (PV+) interneurons and elevated expression of GluN2BRs in hippocampal (HPC) CA1 subregion as an effect of Aβ_1-42_ intracerebroventricular (ICV) infusion**. (**A**) Immunostaining of PV+ interneurons (red) with cell nuclei counterstained (blue) in selected rat brain sections (Bregma -3.80 mm). Left panel: overview of increased PV+ interneurons distribution within the HPC after Aβ_1-42_ ICV (b) *vs.* SCR ICV (a) infusion (scale bar: 200 µm). Right panel: a high-magnification (scale bar: 20 µm) images of the squared areas of HPC CA1 subregion display a higher distribution of PV+ interneurons in *stratum pyramidale* (SP) and *stratum radiatum* (SR) after Aβ_1-42_ ICV (d) *vs.* SCR ICV (c) infusion. Quantification of PV+ interneurons revealed a significant increase in their number in Aβ_1-42_-treated animals within specific HPC subregions. Intergroup statistical differences were evaluated using an unpaired t-test (n=18 per group, *p≤0.05, **p≤0.01, ***p≤0.001, ****p≤0.0001). Data are presented as violin plots with the median (horizontal line), with lower and upper quartile ranges (25-75%, dotted line). (**B**) GluN2BRs immunostaining (red) with counterstained cell nuclei (blue) in the SR of CA1 region reveals increased GluN2BRs signal following Aβ_1-42_ICV infusion (b) compared to SCR-treated controls (a) in selected rat brain sections (Bregma -3.80 mm, scale bar: 20 µm). Differences in average fluorescence intensity in the red channel were evaluated using an unpaired t-test (n=6 per group, *p≤0.05, **p≤0.01, ***p≤0.001, ****p≤0.0001). Data are presented as the median (horizontal line) with lower and upper quartile ranges (25-75%, box) with whiskers for minimum and maximum.

### Lamotrigine enhances the amplitude of hippocampal theta oscillations 14 days post-Aβ_1-42_ ICV infusion *in vivo*

To evaluate the neuromodulatory effects of LTG on local hippocampal networks dynamics and their synchronization capacity, we specifically assessed the impact of a single intrahippocampal LTG injection on hippocampal theta oscillations 14 days post-SCR or Aβ_1-42_ICV infusion, using repeated-measures ANOVA. This time point was selected due to the prominent alterations in theta rhythm observed in our *in vivo* time-dependence studies. Consistent with previous findings, theta amplitude and power significantly decreased after Aβ_1-42_ ICV infusions regardless of the LTG treatment (amplitude: F_(1,8)_=672.48, p≤0.0001; power: F_(1,8)_=586.01, p≤0.0001). We found that LTG administration led to a significant increase in theta amplitude and power across groups (amplitude: F_(1,8)_=192.49, p≤0.0001; power: F_(1,8)_=189.45, p≤0.0001; Fig. 6A). This enhancement was modulated by treatment interaction, with LTG effects on theta amplitude and power varying between SCR and Aβ_1-42_ groups (amplitude: F_(1,8)_=53.08, p≤0.0001; power: F_(1,8)_=137.50, p≤0.0001). *Post hoc* analysis confirmed that both theta amplitude and power remained significantly reduced in Aβ_1-42_-infused animals compared to SCR animals, both with and without LTG treatment (amplitude: SCR+LTG *vs.* Aβ_1-42_+LTG, p≤0.0001; SCR *vs.* Aβ_1-42_, p≤0.0001; power: SCR+LTG *vs*. Aβ_1-42_+LTG, p≤0.0001; SCR *vs.* Aβ_1-42_, p≤0.0001; Fig. 6A).

**Figure 6.**
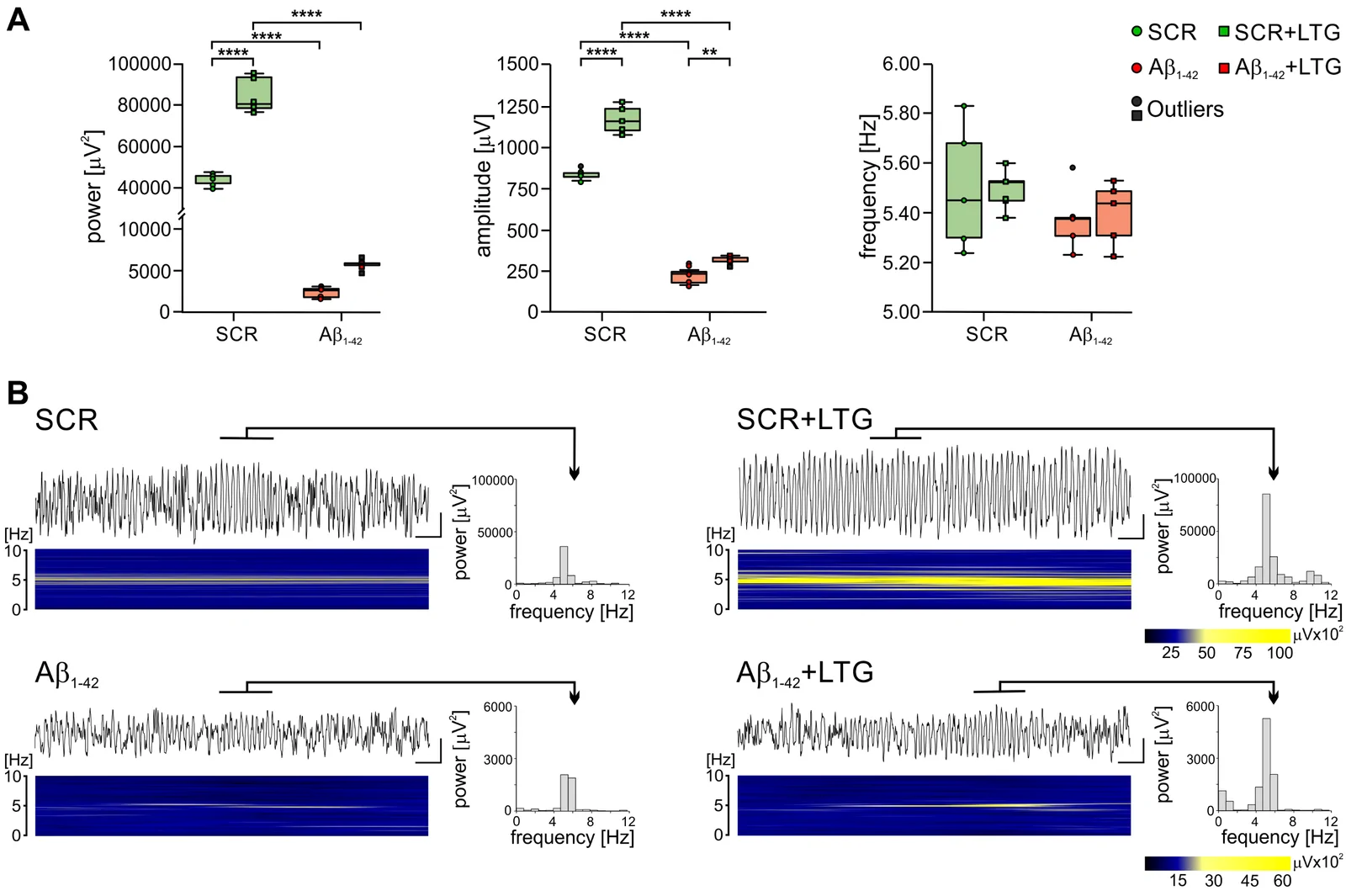
**Augmenting effect of lamotrigine (LTG) on hippocampal (HPC) theta oscillations in anesthetized rats intracerebroventricularly (ICV) infused with scrambled Aβ_1-42_ peptides (SCR) or Aβ_1-42_ oligomers (Aβ_1-42_) (n=5 per group)**. (**A**) Increase in power and amplitude, but not frequency, of HPC theta oscillations after local LTG injection was observed. Data are presented as the median (horizontal line) with lower and upper quartile ranges (25-75%, box) with whiskers for minimum and maximum. Significant differences were assessed using repeated-measures ANOVA followed by the Bonferroni *post hoc* test (*p≤0.05, **p≤0.01, ***p≤0.001, ****p≤0.0001). (**B**) The power-frequency spectrograms were taken from 15 s HPC field potential recordings in rats before (left panel) and after LTG injection (right panel). Arrows indicate FFT histograms calculated from HPC field potential samples (2s) recorded in CA1 field of HPC *in vivo*. Calibration: 1 s and 500 μV for SCR; 1 s and 250 μV for Aβ_1-42_.

Notably, LTG significantly increased theta amplitude and power in SCR animals (amplitude: SCR *vs*. SCR+LTG, p≤0.0001; power: SCR *vs*. SCR+LTG, p≤0.0001), whereas in Aβ_1-42_-infused animals, LTG only increased amplitude (Aβ_1-42_ *vs.* Aβ_1-42_+LTG, p=0.0097) without affecting power (Aβ_1-42_ *vs.* Aβ_1-42_+LTG, p=1.0000; Fig. 6A). Theta frequency remained unaffected by either Aβ_1-42_ or SCR ICV infusions (F_(1,8)_=2.11, p=0.1846) or LTG treatment (F_(1,8)_=0.00, p=0.9662), or their interaction (F_(1,8)_=0.01, p=0.9437; Fig. 6A). Since LTG significantly increased HPC theta amplitude in Aβ_1-42_-treated animals (Fig. 6A-B), it may hold therapeutic potential to counteract Aβ oligomers-induced neuronal synchrony dysfunction.

**Figure 7.**
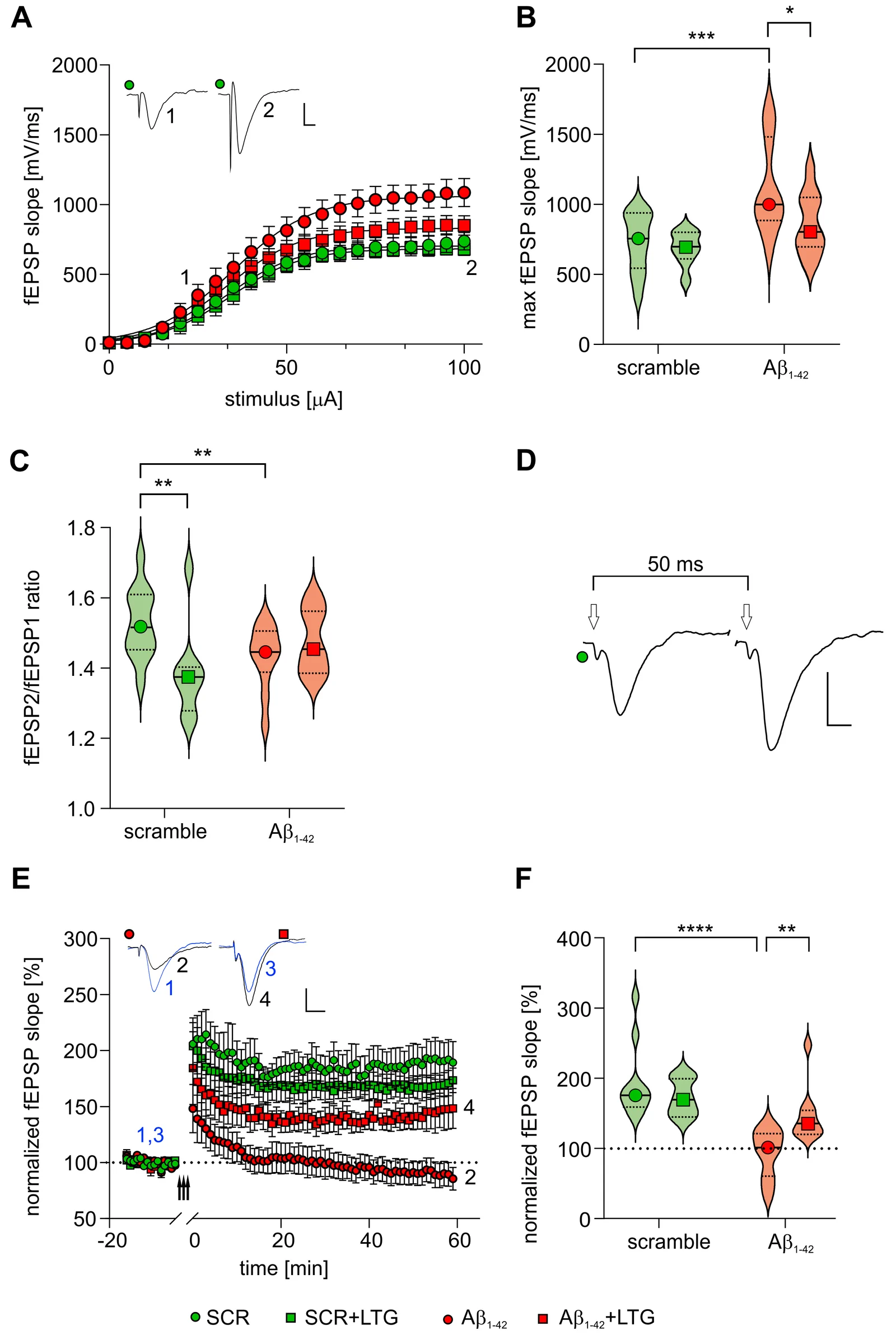
**Normalizing effect of lamotrigine (LTG) on synaptic transmission and LTP impaired by Aβ_1-42_ oligomers, as evidenced by modulation of stimulus intensity-response relationship in the *stratum radiatum* of the CA1 region in hippocampal (HPC) slices**. Data are shown for HPC slices obtained from rats intracerebroventricularly (ICV) infused with scrambled Aβ_1-42_peptide (SCR; n=12), Aβ_1-42_oligomers (Aβ_1-42_; n=10), SCR with 60-min LTG incubation (SCR+LTG; n=8), and Aβ_1-42_with 60-min LTG incubation (Aβ_1-42_+LTG; n=10). (**A**) Plots of the fEPSP slope and stimulation intensity in experimental groups. Insets in A show representative fEPSPs from control rats at two stimulus intensities (1, 2). Black lines represent Boltzmann function fits to the data. (**B**) Comparison of calculated maximal fEPSP slopes in experimental groups. (**C**) Paired-pulse (FP2/FP1) ratio in experimental groups. Insets in (D) show an example of a field potential pair evoked in a representative control (SCR) slice. Arrows mark the stimulus point. (**E**) Plots of the normalized fEPSP slope (mean values) recorded from HPC slices. Arrows denote the time of theta-burst stimulation (TBS, repeated 3 times). Insets in E show superposition of fEPSPs recorded in representative experiments before and after TBS at time points indicated by numbers. (**F**) Normalized fEPSP slope recorded between 45-60 min after TBS, relative to baseline. Data on panels B, C, and F are presented as violin plots with the median (horizontal line) and with lower and upper quartile ranges (25-75%, dotted line). Significant differences were estimated by a two-way ANOVA followed by Tukey’s *post hoc* test (*p≤0.05, **p≤0.01, ***p≤0.001, ****p≤0.0001).

### Lamotrigine restores hippocampal long-term potentiation impairment *in vitro* 14 days post-Aβ_1-42_ ICV infusion

To determine the neuromodulatory effect of LTG on synaptic plasticity, we analyzed the impact of a single intrahippocampal LTG administration on fEPSP slope 14 days post-Aβ_1-42_ ICV infusion, using two-way ANOVA. This time point was selected based on our prior observations of pronounced theta impairments at 14 day *in vitro*, while still allowing for the reliable detection of LTP and potential pharmacological rescue effects. Considering the main effects of the examined treatments (Aβ_1-42_ or SCR ICV infusion and LTG slice incubation) there was no significant interaction between infusion type and LTG administration (F_(1,36)_=2.06, p=0.1602), but there was a significant effect of Aβ_1-42_ ICV infusion on fEPSP slope in comparison to SCR controls (F_(1, 36)_=13.94, p=0.0006; Fig. 7A).

**Figure 8.**
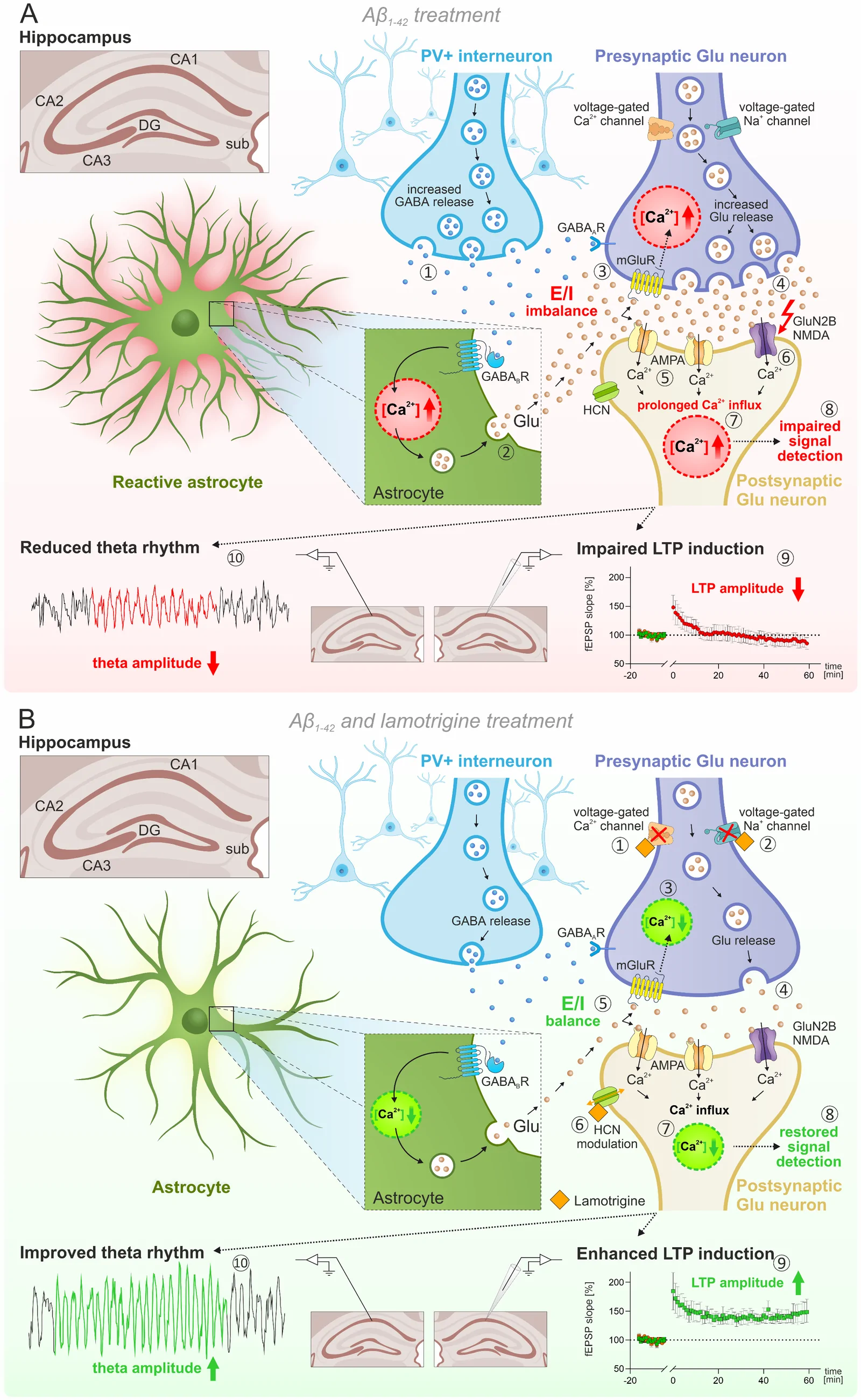
**Simplified model of the probable mechanism of Lamotrigine (LTG)-mediated restoration of the E/I balance disrupted by Aβ_1-42_ oligomers in hippocampal networks**. (**A**) Aβ_1-42_ oligomers initiate inflammation and a cascade of synaptic alterations: elevating intracellular Ca²^+^ levels, promoting glutamate (Glu) release, inhibiting its uptake by activated astrocytes, and triggering a feedback loop contributing to the E/I imbalance. GABA released from interneurons, including PV+ cells (1), activates astrocytic GABA_B_Rs, inducing Ca²^+^-dependent Glu release (2). Glu enhances presynaptic mGluR activation (3), promoting further Glu release (4). Prolonged Glu release activates AMPA receptors (5) and overexcites NMDA GluN2B-containing receptors (6) leading to their decomposition. This causes disrupted/prolonged Ca²^+^ influx to postsynaptic neuron (7), synaptic noise, and impaired signal detection (8). Sustained Ca²^+^ influx *via* dysregulated NMDA receptors results in synaptotoxicity, impaired LTP (9) and reduced theta rhythm (10). (**B**) Lamotrigine likely reverses key components of this pathological cycle, possibly through its anti-inflammatory action and by preventing neuronal hyperexcitability (including PV+ neurons) *via* inhibition of presynaptic voltage-gated calcium (1) and sodium (2) channels, thereby balancing intracellular Ca²^+^ signaling (3) and Glu release (4), ultimately restoring the E/I balance (5). LTG also modulates HCN channels in postsynaptic neurons (6) stabilizing neuronal membrane potential, reducing neuronal excitability and normalizing calcium influx (7) which in turn restores signal detection (8), enhancing LTP induction (9) and theta rhythm (10).

While maximal fEPSPs slope in control animals (SCR group) reached 753.1 (531.4-936) mV/ms (median and quartile range), in slices obtained from rats subjected to previous Aβ_1-42_ ICV infusion (Aβ_1-42_ group) maximal fEPSPs slope was visibly higher [1004 (885.6-1494) mV/ms; p=0.0005; Fig. 7B]. Moreover, a Boltzmann fitting of data collected from HPC slices obtained from Aβ_1-42_ ICV-treated animals and incubated with LTG (Aβ_1-42_+LTG group) showed a significant slope decrease compared to Aβ_1-42_ group [802 (689.2-1050), p=0.0205; Fig. 7B]. Incubation with LTG had no effect on the maximal fEPSP slope in slices obtained from rats previously exposed to SCR ICV infusion [693.8 (601.9-795.9) mV] compared to SCR controls (p=0.9710; Fig. 7B). These results suggest that LTG might counteract Aβ_1-42_-induced neuronal hyperactivity and that LTG is ineffective under control conditions unless there is pre-existing synaptic dysfunction.

ANOVA analysis on the effects of treatments used on paired-pulse response revealed an interaction between previous Aβ_1-42_ ICV infusion and LTG incubation (F_(1, 37)_=8.15, p=0.007) but no significant effect of Aβ_1-42_ ICV infusion (F_(1, 37)_=0.002, p=0.96) or LTG treatment (F_(1, 37)_=2.35, p=0.1342). *Post hoc* data revealed that Aβ_1-42_ ICV infusion as well as LTG treatment decreased the paired-pulse response amplitude ratio (FP2/FP1) [1.34 (1.22-1.37), p=0.0044 and 1.429 (1.36-1.5), respectively] compared to SCR controls [1.51 (1.44-1.62); Fig. 7C], as further illustrated by the example of a field potential pair evoked in a representative control slice (Fig. 7D). Two-way ANOVA of fEPSPs recorded between 45-60 minutes after TBS delivery revealed significant effects of Aβ_1-42_ ICV infusion (F_(1, 31)_=19.32, p=0.0001), but no effect of LTG slice incubation (F_(1,31)_=1.28, p=0.2670) nor their interaction (F_(1,31)_=6.72, p=0.0141) on the magnitude of LTP (Fig. 7E-F). The recorded LTP was significantly reduced in Aβ_1-42_ group compared to the SCR controls [97.74 (58-120) % *vs.* 171.3 (157-200) %, respectively; p≤0.0001] while HPC slice incubation with LTG resulted in a reversal of the effect of Aβ_1-42_ ICV treatment [Aβ_1-42_+LTG; 131.8 (118-152) %, p=0.0087; Fig. 7E-F], suggesting its potential to counteract Aβ_1-42_-induced synaptic impairments. LTG treatment had no effect on LTP magnitude in SCR controls [165.1 (143-198) %, p=0.3351; Fig. 7E-F], indicating that its neuromodulatory effects are specific to pathological states rather than general synaptic enhancement.

## DISCUSSION

In this study we show that Aβ_1-42_ ICV infusions led to a progressive, time-dependent disruption of HPC theta oscillations, evidenced by marked reductions in amplitude and power, both *in vivo* and *in vitro*. Aβ_1-42_ ICV infusions also resulted in the abolishment of LTP in hippocampal CA1 *stratum radiatum*, evoked by stimulation of Schaffer collaterals *in vitro*. This electrophysiological impairment was accompanied by significant histopathological changes observed 14 days post-infusion, including enlarged lateral ventricles, tau protein hyper-phosphorylation (CA1), neuronal loss, reactive astrogliosis, characterized by both astrocytic hypertrophy and increased astrocyte density (CA1-CA3, DG, and *subiculum*), PV+ increased density (CA1-CA3, *subiculum*) and upregulation of GluN2BRs in CA1 *stratum radiatum*, suggesting widespread HPC network dysfunction. Notably, LTG partially restored both theta amplitude as well as LTP, highlighting its neuromodulatory and neuroprotective effects in counteracting early Aβ-induced synaptic deficits and network alterations.

Our results are in line with earlier studies indicating that acute administration of Aβ oligomers disrupts HPC network activity, reducing spontaneous synaptic activity, subthreshold oscillations, and Ca²^+^ transients in CA1 pyramidal neurons [49]. These effects were associated with disrupted network dynamics and synaptic transmission, likely due to changes in ionic currents such as I*h*. Synaptic dysfunction included both presynaptic changes (increased paired-pulse facilitation) and postsynaptic deficits (reduced LTP amplitude) that reflected the toxic effect of Aβ oligomers. Notably, following intracerebral infusion, Aβ_1-42_undergoes a time-dependent aggregation process *in vivo*, transitioning from monomers to small oligomers (e.g., dimers and trimers), then to larger oligomeric assemblies, protofibrils, and eventually fibrils. This process depends on multiple factors, including the infusion site, local diffusion, and peptide clearance [82-85]. Our findings are consistent with the time-dependent synaptotoxicity of soluble Aβ_1-42_oligomers, which have been shown to impair hippocampal plasticity and memory processes independently of the detection of fibrillar aggregates [83, 86]. The gradual decrease in detectable Aβ_1-42_immunoreactivity up to 14 days post-infusion suggests a temporal dissociation between the presence of amyloid in the tissue and the persistence of functional impairments. This aligns with reports of rapid Aβ clearance from the brain parenchyma *via* phagocytic mechanisms, as demonstrated by Nakagawa et al. [87], who observed near-complete removal of aggregated Aβ_1-42_from the ventricular system within 28 days post-infusion. Extending these observations, ICV infusions of Aβ_25-35_ oligomers impaired learning in rats within three days post-infusion, correlating with a failure of HPC neurons to generate membrane potential oscillations (MPOs) [88], which underlie local theta generation [89, 90]. Kalweit et al. [91] showed that intrahippocampal Aβ_1-42_ infusions altered theta and gamma oscillations 24 h post-infusion. Building on previous work, our findings provide the first evidence of a gradual loss of theta oscillations, indicating a progressive decline in the HPC network’s capacity for synchronized activity. This suggests a substantial breakdown in synaptic integrity and interneuronal communication, ultimately impairing the ability to sustain coordinated theta rhythms essential for proper cognitive function. Although we did not perform behavioral assays showing learning deficits associated with theta alteration, there is a long and strong literature that has repeatedly demonstrated this [92, 93]. Interestingly, we observed a marked reduction in theta amplitude and power after ICV Aβ_1-42_ infusions, while frequency remained largely unaffected *in vivo*. In contrast, a decline in frequency was evident in hippocampal slices, supporting the idea that amplitude is primarily shaped by local HPC network performance, whereas frequency is governed predominantly by ascending septal inputs [46].

Earlier reports that used Aβ_1-42_ infusions bilaterally into older mice found that the hippocampal CA1 region was the first area to exhibit pathological changes during AD progression [94], resulting in significant localized upregulation of pTau and GFAP between days 7 and 30, accompanied by cognitive deterioration. Our histological findings, indicating neurodegeneration, are consistent with previous reports showing early AD-related neuronal loss primarily in the DG, CA3 thorny excrescences, and dendrites of CA1 pyramidal neurons. This supports the idea that the EC layer II → DG → CA3 → CA1 circuit is especially vulnerable to early degeneration [94], potentially underlying the progressive disruption of theta rhythm observed in our study.

Hyperphosphorylated Tau contributes to channelopathies, including dysfunction of HCN channels, and to synaptic impairments [95], and induces inflammatory responses and cell death [96], resulting in memory disturbances involving multiple mechanisms. Increased Tau phosphorylation is one of the earliest events in AD development [97] and is essential for Aβ-induced impairment of LTP induction in HPC [98]. Previous studies have demonstrated that principal cells and PV+ interneurons in adolescent AD mouse models accumulate pTau at microtubule domain sites, which correlates with HPC theta rhythm impairments and general excitability alterations in CA1, supporting our idea of theta as a potential biomarker of early AD progression [99-101]. Aberrant translation and redistribution of pTau to the somatodendritic compartment can result from overstimulation of AMPA and NMDA receptors [102]. Alternatively, Aβ_1-42_ oligomers may trigger pTau translocation from axons to dendritic spines, leading to early synaptotoxicity, spine loss, and excitotoxicity [76, 103]. *Via* NMDAR-mediated Ca²^+^ influx, Aβ_1-42_ oligomers can activate AMPA receptor-activated kinase (AMPK), thereby increasing pTau levels and exacerbating dendritic spine loss [77]. Additionally, pTau may directly enhance Fyn kinase activity, leading to phosphorylation of the NMDAR subunit GluN2BRs and promotes Ca²^+^-dependent excitotoxic signaling [76, 77]. Aβ-induced activation of Tau kinases has also been implicated in excitotoxic processes [38, 76, 100].

Aβ oligomers may disrupt hippocampal oscillatory activity by altering GABAergic interneurons, including PV+ cells, beyond their effects on cholinergic and glutamatergic pathways [104, 105]. In the early stage of AD, soluble Aβ induces hyperexcitability in PV+ interneurons, contributing to network dysfunction and cognitive decline, while suppression of PV+ activity can restore memory by reducing excessive inhibition of pyramidal cells [40, 106, 107]. These effects stem from an imbalance in inhibitory circuitry, particularly involving PV+ GABAergic interneurons that provide feedforward and feedback inhibition critical for coordinating HPC oscillations [7, 37, 39]. PV+ interneurons are essential for theta-band synchronization and theta-based temporal coding in HPC principal cells [108, 109]. Their role in intrinsic theta generation has been demonstrated through *in vitro* and computational models [110, 111], as well as *via* NMDAR deletion studies disrupting CA1 theta oscillations [112]. PV+ fast-spiking interneurons receive excitatory inputs from superficial pyramidal neurons and inhibit deep-layer pyramids, contributing to the E/I balance [113]. They also target other interneurons and contribute to fine-tuned regulation of network dynamics. Tonic inhibition, mediated *via* extrasynaptic GABA_A_ receptors, controls both pyramidal cell excitability and interneuron activity [37, 114]. Notably, low extracellular GABA levels depolarize PV+ cells, enhancing inhibition, whereas high GABA- such as in Aβ_1-42_ pathology - causes hyperpolarization and disinhibition [115]. *In vitro* recordings confirm increased tonic GABAergic conductance in CA1 after Aβ_1-42_ infusion [32]. GABAergic dysfunction in PV+ interneurons is well documented in AD. Structural degeneration, including reduced soma and axon size, and >70% loss of PV+ cells have been reported in HPC postmortem tissue [116]. However, cortical data remain inconsistent [117, 118], suggesting region- or subtype-specific variability. Similarly, young AD mouse models exhibit reduced theta rhythmicity due to decreased PV+ intrinsic excitability, and restoring PV+ activity may recover network synchrony [99]. Our observation of increased PV+ cell numbers in the CA1 region following Aβ_1-42_ infusion may reflect a compensatory response to Aβ_1-42_-mediated dysfunction in oscillatory regulation. This finding aligns with the idea that PV+ interneuron involvement varies with AD subtype (familial AD - fAD *vs*. sAD) and disease stage (early *vs*. late). For example, Ali et al. [119] reported PV+ cell loss in CA1 and CA2 in 5xfAD mice and highlighted heterogeneity across HPC sublayers. Similarly, Zheng et al. [120] found pTau accumulation in DG PV+ interneurons of AD patients and triple transgenic (3xTg) mice expressing mutant human APP, PS1, and Tau genes, accompanied by astrogliosis, impaired neurogenesis, and local hyperactivation of excitatory neurons. Possibly, our findings align with the idea of Hijazi et al. [106] that early hyperexcitability of PV+ interneurons may create a vulnerable network state, predisposing HPC to progressive dysfunction and AD-like neuronal alterations, as reflected in the disrupted theta oscillations. Previous studies have shown that both significant increases [121] and decreases [122] in parvalbumin protein expression, as well as in the number of PV+ interneurons, can be observed in rat brains due to alterations in cell signaling [123] or in response to factors such as physical exercise or specific types of neuronal stimulation [124]. Given the intrinsic plasticity of interneurons, particularly GABAergic subtypes, which can dynamically regulate the expression of markers such as PV in response to changes in neuronal network activity or molecular milieu, our findings support the interpretation that Aβ_1-42_ oligomer exposure induced a genuine upregulation of PV expression in pre-existing hippocampal interneurons.

Busche et al. [45] showed that Aβ_1-42_ application in wild-type mouse cortex increased oscillation frequency and disrupted coherence, suggesting an E/I imbalance. These effects were linked to elevated Ca²^+^ transients [125]. Studies of AD progression indicate that a reduction of 30% or more in Ca^2+^-dependent conductance leads to irregular spike generation in pyramidal neurons, resulting in irregular theta-band activity and highlighting calcium's role in AD-related neural dysfunction [126, 127]. Aβ_1-42_ promotes Ca²^+^-permeable AMPAR incorporation *via* AKAP-CaN signaling, which later removes both Ca²^+^-permeable and -impermeable AMPARs, blocking LTP [128]. Theta rhythms are associated with LTP induction, with interneurons contributing to Hebbian LTP through mGlu receptor activation and Ca²^+^ signaling [37]. The maximal slope of EPSPs may increase following excitation due to enhanced glutamate release induced by the Aβ_1-42_ infusion. This could affect metaplasticity, the activity-dependent modulation of synaptic plasticity, where prior synaptic activity alters the ability of synapses to undergo subsequent plastic changes. Excessive excitation leads to a state of hyperactive tissue, which, following tetanization, becomes unresponsive to subsequent stimuli, thereby preventing LTP induction or resulting in a diminished LTP response, as observed in our study.

Aβ oligomers were shown to reduce synaptic transmission *via* NMDARs [129] and impair glutamate clearance, contributing to excitotoxicity in AD [130]. Since NMDARs are critical for cholinergic HPC theta modulation [47, 131, 132], findings by Gutiérrez-Lerma et al. [58] are notable, demonstrating that bath application of Aβ oligomers disrupts both carbachol- and dihydroxyphenylglycine-induced theta rhythms in HPC slices. Yi et al. [133] proposed that Aβ oligomers weaken the function of the muscarinic acetylcholine receptors (mAChRs) subtype M1 *via* mGluR5-mediated mechanisms, destabilizing mAChRs. Dysfunction of these receptors was found to depend on Ca²^+^ mobilization and NMDARs activity, possibly through mGluR5 or its interaction with NMDARs. Notably, M1AChRs are key regulators of synaptic signaling pathways and theta rhythm generation [134]. Akay et al. [135] showed impaired theta generation in slices from triple-transgenic AD mice treated with carbachol, while Janíčková et al. [136] revealed that Aβ disrupts M1AChRs/G-protein coupling. These findings raise the question of whether the GluN2BR overexpression/redistribution in CA1 observed in our study represents a consequence of Aβ pathology, or a compensatory mechanism.

Previous studies reported LTP and learning deficits a few days after intracerebral Aβ_1-42_ treatment [137, 138], possibly due to elevated extrasynaptic glutamate enhancing GluN2BRs activation and mediating hyperexcitability. Südkamp et al. [78] highlighted the roles of GluN2A and GluN2B receptors in HPC excitability and LTP following Aβ_1-42_ injection in aging mice. Aβ_1-42_-induced LTP impairment in CA1, observed in HPC slices, was fully reversed by GluN2BRs negative allosteric modulators [139]. However, another study found that Aβ_1-42_ reduced LTP without affecting excitability in wild-type mice, an effect absent in GluN2A+/- and GluN2B+/- mice, suggesting that LTP deficits may occur independently of GluN2A/GluN2B receptors. Varga et al. [75] showed that Aβ_1-42_-induced hyperexcitability and LTP impairment (which is in line with our results) required GluN2BRs activation at extrasynaptic sites, as blockade of GluN2BRs prevented these effects. We observed increased GluN2BRs levels in the CA1 *stratum radiatum* 14 days post-infusion. Similarly, Ortiz-Sanz et al. [140] reported GluN2BRs overexpression in postsynaptic density-enriched fractions from AD patients at early disease stages (Braak II), correlating with Aβ load. They found that intrahippocampal Aβ_1-42_oligomers induced GluN2BRs upregulation in the DG and increased NMDAR-dependent Ca²^+^ influx *via* GluN2BRs phosphorylation and integrin β1/PKC signaling. Further research is needed to determine whether these changes represent early compensatory responses or pathological NMDARs dysregulation leading to excitotoxicity.

HCN channels, regulated by Ca^2+^ are essential for dendritic integration, synaptic plasticity, and memory formation [62, 90, 141-143]. Dendritic HCN channels modulate neuronal integrative properties and contribute to rhythmogenesis and subthreshold MPOs [144, 145]. In Tau35 mice, neurodegeneration is marked by pTau, increased HCN expression, dendritic trimming, synaptic loss, and vesicle clustering defects in CA1 [95]. Lamotrigine modulates HCN channels, reduces pacemaker current and slows neuronal discharge [62, 146]. Lamotrigine reduces Ca²^+^-dependent excitability and glutamate release [69], modulates intracellular Ca²^+^ signaling by inhibiting its’ release and efflux [146], and exhibits neuroprotective effects [62]. Chronic treatment with LTG suppresses abnormal spike activity and thus restores LTP, prevents neuronal loss, suppresses Aβ generation and attenuates astrocyte activation induced in APP/PS1 mice [60]. We applied a single intra-hippocampal LTG administration 14 days post-Aβ_1-42_infusion to target a window of maximal, yet reversible theta disruption. This localized approach builds on our earlier *in vivo* and *in vitro* findings demonstrating that intrahippocampal LTG administration enhances theta amplitude, an effect abolished by the HCN blocker ZD7288, indicating that its impact on hippocampal network synchronization involves I*h* current modulation [73] consistent with previous literature showing direct effects of LTG on HCN channels [147-149].

Astrocyte-microglia interaction may be crucial in early AD, and its disturbance could accelerate neurodegeneration and impair memory in the 5xfAD mouse model [12]. Astrocytes are both GABA-ceptive and GABA-ergic cells [12, 150], and in middle-aged AD mice, they increase in size, excessively accumulate and release GABA-even without amyloidosis [10]. Abnormal GABA levels have also been observed in AD patient samples [151]. Astrocytes represent a heterogeneous population and effects of GABA on astrocyte-regulated inflammatory responses are highly dynamic and bring conflicting observations [150]. A reduction in GABA_A_Rs-mediated slow currents in DG, similar to Aβ-induced alterations in CA1 neurons, may disrupt early HPC processing and contribute to the elevated E/I ratio observed in AD patients and animal models [152, 153]. Interestingly, Aβ oligomers induce hyperactivity in CA1 neurons by impairing astroglial glutamate reuptake *via* reduced expression and membrane mobility of excitatory amino-acid transporter 2EAAT2/GLT-1, thereby enhancing excitatory transmission [154, 155]. A possible mechanism that could account for the effect observed in our study is that increased level of Aβ_1-42_ oligomers and pTau initiates a cascade of synaptic alterations elevating intracellular Ca^2+^ levels, promoting glutamate release, inhibiting its uptake by astrocytes, and establishing a feedback loop that contributes to E/I imbalance. Activated astrocytes, acting as both GABA-ceptive and GABA-ergic cells, respond by modulating glutamatergic transmission. GABA released from interneurons, including PV+ cells, suppresses presynaptic glutamate release, while GABA_B_Rs activation on astrocytes triggers Ca²^+^-dependent glutamate release, enabling astrocytes to transform inhibitory signals into excitatory output [156]. This astrocyte-derived glutamate enhances presynaptic mGluR activation, further promoting glutamate release, and also acts on postsynaptic neurons to counteract GABAergic inhibition [12]. This cycle leads to aberrant neuronal activity, glutamatergic hypersynchronization, and elevated extracellular glutamate, particularly affecting excitatory synapses, promoting network hyperactivity [20, 157]. Prolonged glutamate accumulation activates NMDA and AMPA receptors, leading to their internalization and/or redistribution, especially of NMDARs. Altered membrane composition promotes overactivation of extrasynaptic GluN2B-containing receptors, disrupting Ca^2+^ influx essential for LTD and impairing hippocampal LTP [20, 158]. This tonic overexcitation prevents Mg^2+^ from its’ filtering function, increasing synaptic noise by prolonged moderate Ca^2+^ influx, impairing signal detection [159]. As a result, sustained Ca²^+^ influx through a dysregulated NMDAR conductance ultimately could lead to the synaptotoxicity, impaired LTP and reduced theta oscillations observed in our study (Fig. 8A). Our data suggest that LTG may reverse key components of this pathological mechanism, possibly by balancing intracellular Ca^2+^ signaling, glutamate-driven hyperexcitability, and HCN channel modulation, thereby restoring network synchrony and plasticity (Fig. 8B).

## Limitations of the study

Our study offers new insights into early Aβ_1-42_-induced network dysfunction in a rat model of sAD, identifying theta rhythm disruption as a potential electrophysiological biomarker and highlighting lamotrigine as a promising neuroprotective and restorative agent. Nonetheless, several limitations should be noted. We did not evaluate HCN channel expression, intracellular Ca^2+^ levels, or specific markers such as GFAP and GluN2BRs using molecular quantification techniques. Instead, we relied on semi-quantitative immunofluorescence, which, while informative, lacks the molecular specificity of direct protein quantification. Future research could benefit from histological verification following LTG treatment and inclusion of LAMP5-positive interneuron subtypes, such as ivy and neurogliaform cells, given their potential contribution to GABA_A_Rs slow-mediated theta oscillations and AD pathology [160].

## Conclusions

Our findings reveal that early disruptions in hippocampal theta oscillations, triggered by Aβ_1-42_ oligomers represent a sensitive biomarker of preclinical network dysfunction and neurodegeneration. This pathology is accompanied by Tau hyperphosphorylation, impaired synaptic plasticity, increased PV+ density, and astrogliosis all occurring before detectable amyloid plaque deposition. We propose a direct cause-and-effect relationship in which early fluctuations and progressive loss of hippocampal theta rhythms, triggered by Aβ_1-42_ oligomers may result from impaired HCN channel function, increased glutamate release and disrupted Ca^2+^-dependent excitability. These oscillatory deficits reflect a breakdown in the temporal coordination of synaptic signal processing, essential for memory encoding and retrieval. As Aβ_1-42_ oligomers further amplify glutamate-driven Ca²^+^ overexcitation, E/I imbalance worsens, degrading theta-generating circuits. This progressive theta disruption marks a functional shift from reversible synaptic dysfunction to irreversible failure, ultimately driving cognitive decline in the dementia process. Importantly, LTG partially restored oscillatory synchrony and LTP, suggesting its potential as an early-stage therapeutic intervention. These results highlight the clinical relevance of targeting HCN channels and Ca²^+^-dependent excitability in Aβ-induced hippocampal dysfunction. Clarifying the role of HCN channels, Ca^2+^ dynamics and molecular mechanisms of LTG action in modulating intra- and interneuronal activity may pave the way for strategies aimed at preventing or delaying cognitive decline in AD, especially in its sporadic form. To this end, future studies should include behavioral assays to correlate electrophysiological alterations with cognitive performance, investigate clinically applicable routes of LTG administration, and examine broader oscillatory dynamics (e.g., global EEG) across multiple frequency bands.

## Supplementary Materials

The Supplementary data can be found online at: www.aginganddisease.org/EN/10.14336/AD.2025.0796.
