# Supplementary material for: Lamotrigine Restores Impairments in Theta Rhythms and LTP as Early Biomarkers of Aβ1-42-Induced Hippocampal Network Dysfunction
Source: Aging Dis. 2026 Oct 4;17(6):3401–26. doi: 10.14336/AD.2025.0796 (PMC13653730; doi:10.14336/AD.2025.0796)
Supplement: Supplementary file 1 [file AD-17-6-3401-s.pdf]

SUPPLEMENTARY DATA

Lamotrigine Restores Impairments in Theta Rhythms and LTP as Early Biomarkers of Aβ_{1-42}-Induced Hippocampal Network Dysfunction

Maciej Studzian, Bartosz Bobula, M. Bruce MacIver, Renata Bocian, Anna Kobrzycka, Bartosz Caban, Tomasz Kowalczyk, Jacek Grebowski, Sylwia Michlewska, Maciej M. Jankowski, Krzysztof Tokarski, Paulina Kazmierska-Grebowska

© 2025. Studzian M lished online at http://www.aginganddisease.org/EN/10.14336/AD.2025.0796

SUPPLEMENTARY DATA

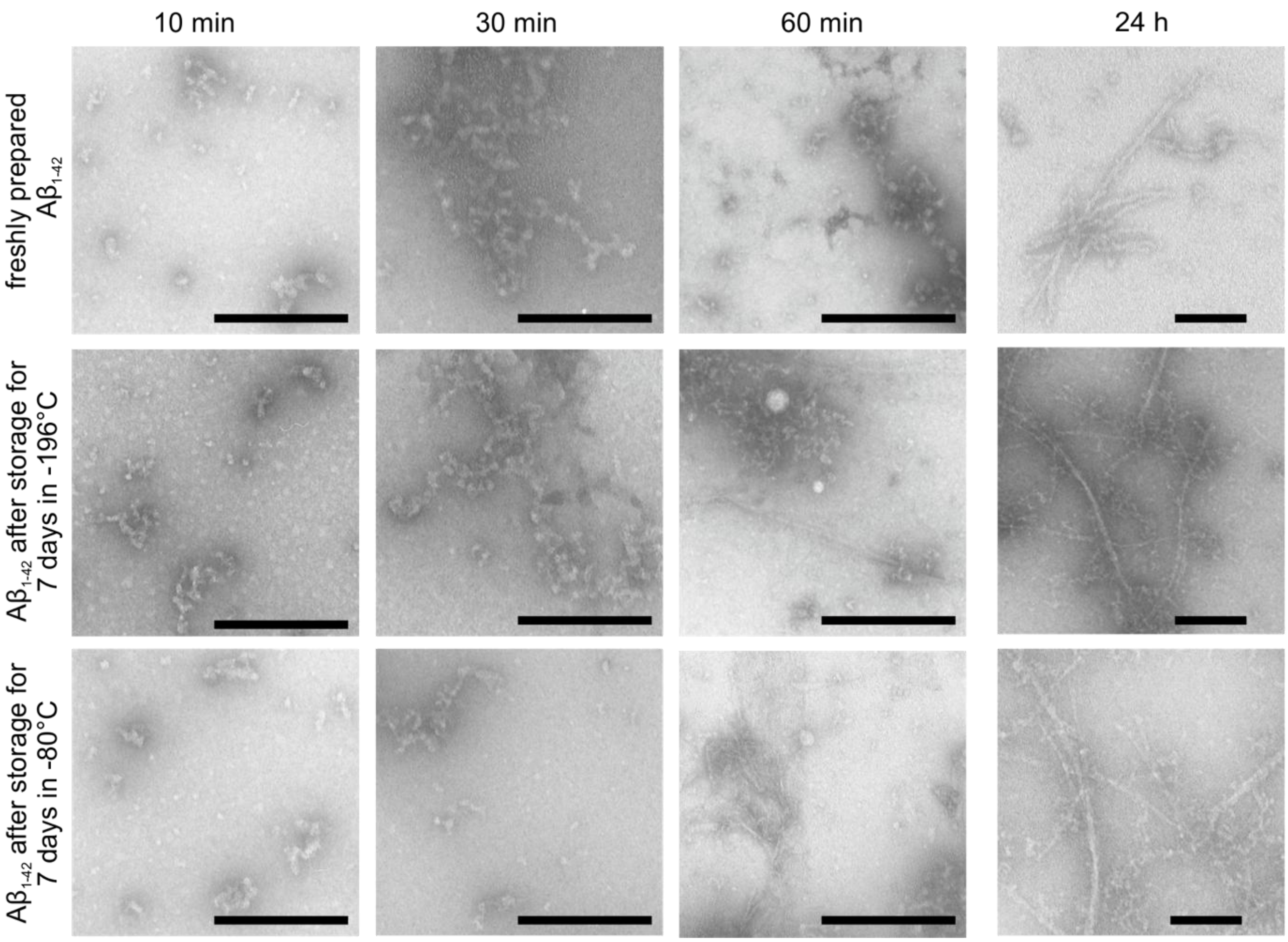

Supplementary Figure 1. Transmission electron microscopy (TEM) images demonstrating the oligomerization of Aβ_{1-42} peptide. Peptide samples were analyzed at multiple time points following resolubilization or thawing from storage at −80 °C or in liquid nitrogen (−196 °C). Samples stored in liquid nitrogen exhibited morphology comparable to that of freshly prepared peptides; therefore, this condition was chosen for subsequent experiments. Scale bar: 200 nm.

© 2025. Studzian M lished online at http://www.aginganddisease.org/EN/10.14336/AD.2025.0796

SUPPLEMENTARY DATA

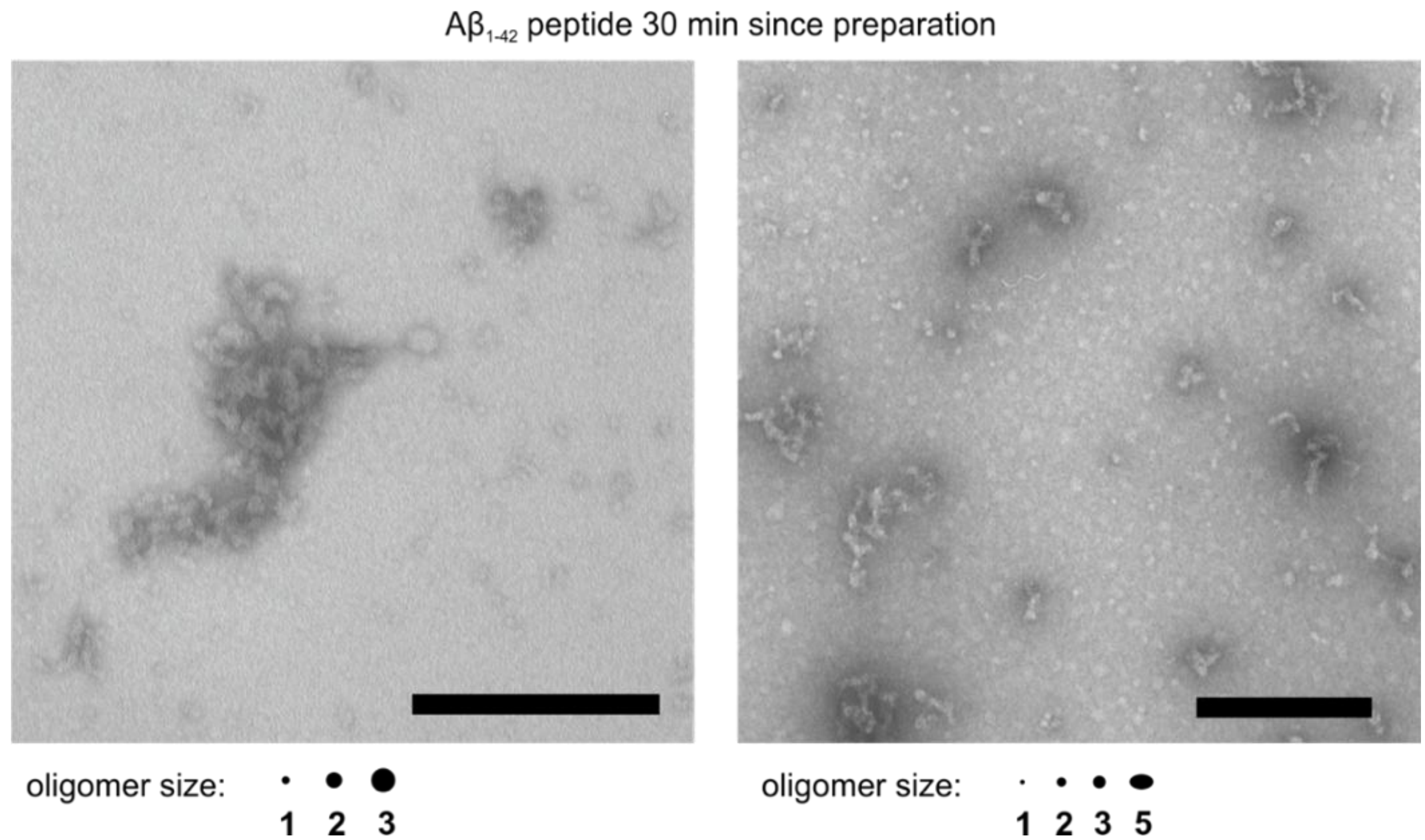

Supplementary Figure 2. Transmission electron microscopy (TEM) images of toxic Aβ_{1-42} peptide oligomers acquired 30 minutes after preparation. Oligomer size was estimated based on an assumed Aβ monomer diameter of approximately 5 nm. Scale bar: 200 nm.

© 2025. Studzian M lished online at http://www.aginganddisease.org/EN/10.14336/AD.2025.0796

SUPPLEMENTARY DATA

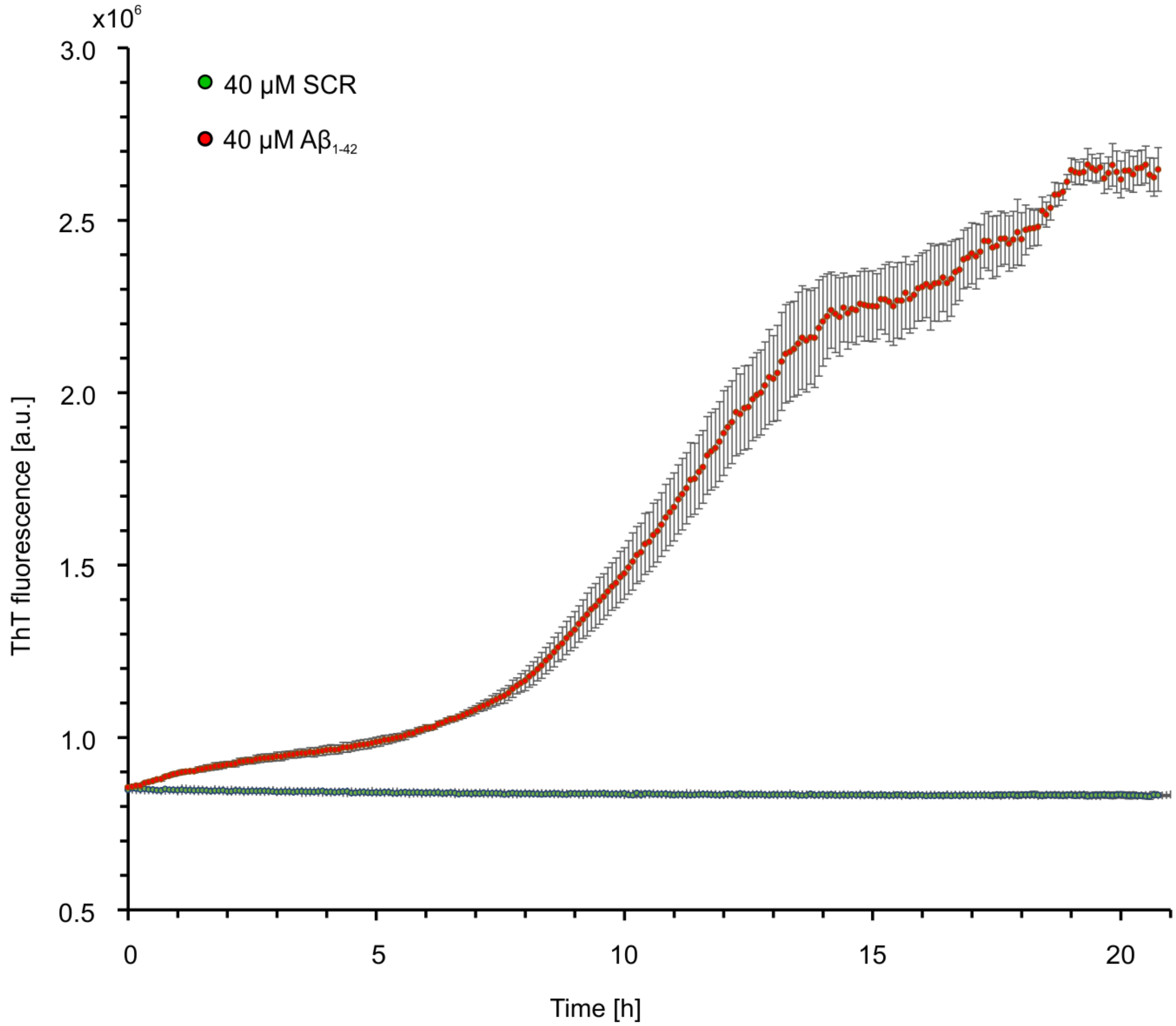

Supplementary Figure 3. Aggregation of $A\beta_{1-42}$ peptides monitored by thioflavin T (ThT) fluorescence. Scrambled peptide (SCR) served as a negative control. ThT was added to peptide samples at a final concentration of 12 µM immediately after resolubilization following HFIP treatment. Data are presented as mean ± SEM (n=3). The most toxic, short oligomeric forms of $A\beta_{1-42}$ are present up to 7 hours post-solubilization. Between approximately 7–14 hours, longer fibrils begin to form, followed by the appearance of insoluble aggregates, as indicated by increased variability in ThT fluorescence measurements.

© 2025. Studzian M lished online at http://www.aginganddisease.org/EN/10.14336/AD.2025.0796

SUPPLEMENTARY DATA

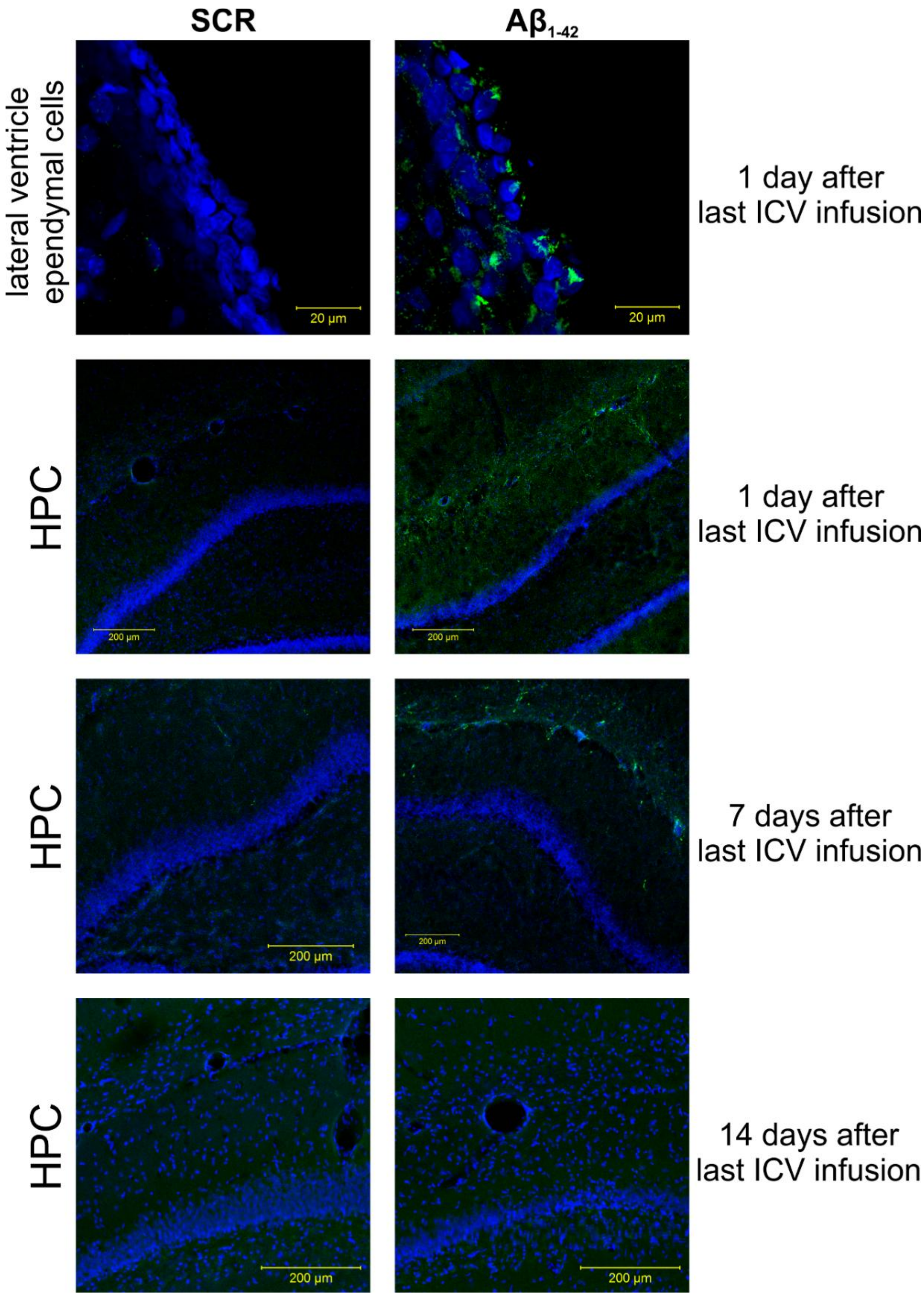

Supplementary Figure 4. Immunostaining of Aβ_{1-42} peptide (green) with cell nuclei counterstained (blue), in selected rat brain sections encompassing the hippocampus (HPC), collected at 1, 7, and 14 days following the final intracerebroventricular (ICV) infusion of scrambled peptide (SCR; negative control) or Aβ_{1-42} oligomers.

© 2025. Studzian M lished online at http://www.aginganddisease.org/EN/10.14336/AD.2025.0796

SUPPLEMENTARY DATA

Supplementary Table 1. Antibodies used in the study.

Antibody	Source	Identifier
Mouse monoclonal anti-phospho-S396 Tau	Cell Signalling Technology	Cat# 9632; RRID: AB_2266237
Mouse monoclonal anti-NeuN	Cell Signalling Technology	Cat# 94403; RRID: AB_2904530
Mouse monoclonal anti-GFAP	Millipore	Cat# MAB360; RRID: AB_11212597
Rabbit monoclonal anti-beta Amyloid 1-42	Abcam	Cat# ab201060; RRID: AB_2818982
Mouse monoclonal anti-PV	Abcam	Cat# ab277625
Rabbit monoclonal anti-NMDAR2B	Abcam	Cat# ab254356; RRID: AB_2895296
Goat anti-Mouse IgG1, Alexa Fluor 488	Thermo Fisher Scientific	Cat# A-21121; RRID: AB_2535764
Goat anti-Mouse IgG2a, Alexa Fluor 594	Thermo Fisher Scientific	Cat# A-21135; RRID: AB_2535774
Goat anti-Mouse IgG2b, Alexa Fluor 594	Thermo Fisher Scientific	Cat# A-21145; RRID: AB_2535781
Goat anti-Rabbit IgG, Alexa Fluor 594	Thermo Fisher Scientific	Cat# A-11037; RRID: AB_2534095

© 2025. Studzian M lished online at http://www.aginganddisease.org/EN/10.14336/AD.2025.0796
